# Impact of the colistin ban on antimicrobial use and resistance-related practices among layer farmers in Indonesia: a comparative KAP study

**DOI:** 10.3389/fvets.2026.1885994

**Published:** 2026-09-23

**Authors:** Mohammad Jokar, Sunandar Sunandar, Annisa Rachmawati, Soe Yu Naing, Happy Nanda Larasati, Ridvana Dwibawa Darmawan, Imron Suandy, Riana Aryani Arief, Nofita Nurbiyanti, Anis Karuniawati, Jaap A. Wagenaar, Diego B. Nobrega, Mathieu Pruvot

**Affiliations:** 1 Faculty of Veterinary Medicine, University of Calgary, Calgary, AB, Canada; 2 O’Brien Institute of Public Health, University of Calgary, Calgary, AB, Canada; 3 AMR—One Health Consortium, Calgary, AB, Canada; 4 PAHO/WHO Collaborating Centre for Research on Antimicrobial Resistance and Appropriate Use of Antimicrobials, University of Calgary, Calgary, AB, Canada; 5 Center for Indonesian Veterinary Analytical Studies (CIVAS), Bogor, Indonesia; 6 Division of Infectious Diseases and Immunology, Faculty of Veterinary Medicine, Utrecht University, Utrecht, Netherlands; 7 WHO Collaborating Center for Reference and Research on Campylobacter and Antimicrobial Resistance from a One-Health Perspective/WOAH Reference Laboratory for Campylobacteriosis, Utrecht, Netherlands; 8 Directorate General of Livestock and Animal Health Services, Ministry of Agriculture, Jakarta, Indonesia; 9 Department of Microbiology, Faculty of Medicine, Universitas Indonesia, Jakarta, Indonesia

**Keywords:** antimicrobial resistance, antimicrobial use, colistin ban, knowledge and practices, poultry

## Abstract

**Background:**

Antimicrobial resistance (AMR) is a growing global public and animal health threat, to which the misuse of antimicrobials in humans and livestock contributes. Here we describe the knowledge, attitudes, and practices (KAP) of Indonesian layer farmers regarding AMR and antimicrobial use (AMU) before the national ban on colistin use, as well as the awareness of farmers of the ban.

**Materials and methods:**

The first phase, conducted in 2014 as part of a study carried out between 2013 and 2016, involved a cross-sectional survey of small-scale layer poultry farmers, assessing their KAP toward AMU and AMR through a structured questionnaire. Farmers’ responses were scored to determine KAP levels. The second study, conducted in 2023 among Indonesian layer farmers following the 2019 colistin ban, used a survey to evaluate 55 participants’ awareness of the ban and their current AMU practices.

**Results:**

Before the colistin ban, 27.5% of layer farmers in Indonesia demonstrated a high level of knowledge regarding AMU and AMR, while the majority had moderate or low knowledge levels. Similarly, only 30% showed a positive attitude, and just 25% exhibited good practices related to AMU and AMR. Tetracyclines, fluoroquinolones, and macrolides were the most commonly used antimicrobials before the ban. After the ban, the use of colistin ceased, but no statistically significant changes in the usage profile of any other antimicrobial were observed. The post-ban survey revealed that 18.2% of the farmers were aware of the colistin ban, and none of the sociodemographic characteristics investigated were associated with awareness levels.

**Conclusion:**

The findings highlight that, although the ban successfully eliminated colistin use in poultry production in Indonesia, limited KAP related to AMR and AMU may become critical bottlenecks to advancing future stewardship strategies.

## Highlights

- In Indonesia, layer poultry farmers generally had moderate scores in knowledge and attitudes toward antimicrobial resistance and antimicrobial use before the colistin ban, with a low proportion demonstrating high scores in practices.
- Farmers commonly reported using tetracyclines and fluoroquinolones, along with macrolides, aminoglycosides, beta-lactams, and colistin. Following the ban, no farms reported using colistin, and the use of other antimicrobials did not change significantly.
- Awareness of the colistin ban after implementation was low, with only 18.2% of respondents reporting knowledge of it.

## Introduction

Poultry farming is a major component of Indonesia’s agricultural economy, as it enhances food security, provides jobs, and generates income (1). Poultry production in Indonesia relies heavily on antimicrobials for disease prevention and treatment (2). In layer poultry farming, antibiotics are frequently used to sustain egg production, prevent bacterial infections, and treat clinical diseases such as colibacillosis, salmonellosis, and mycoplasmosis, which can significantly impact flock health and productivity (3, 4). Among these antibiotics, colistin was commonly used in poultry for prophylactic and therapeutic purposes against Gram-negative bacterial infections; however, because colistin is classified as a last-resort antibiotic for treating multidrug-resistant infections in humans, its misuse in poultry represents a significant public health concern. Recent evidence from South and Southeast Asia, including India, Pakistan, Bangladesh, Nepal, Vietnam, Malaysia, and Indonesia, indicates that poultry and poultry-derived *Escherichia coli* frequently carry phenotypic colistin resistance and/or mobile colistin-resistance genes such as *mcr*-1, with some studies reporting high levels of multidrug resistance and potential dissemination through food-chain or farm-environment (5–8).

Antimicrobial use (AMU) in livestock contributes to antimicrobial resistance (AMR) emergence in animals, with genomic evidence increasingly supporting possible transmission to humans via food, direct contact, and shared environments (9). The European Union has implemented a comprehensive ban on the use of antimicrobials as growth promoters, backed by robust regulatory enforcement, in contrast to many low- and middle-income countries where such practices remain widespread and oversight is limited (10, 11). Although many countries have introduced restrictions or bans on antimicrobial growth promoters, the effectiveness of these policies is often undermined by weak enforcement, limited awareness among farmers, and continued access to antimicrobials through informal or poorly regulated channels (12, 13). For example, in Nepal, despite restrictions on antimicrobial-enriched feed imports, weak surveillance and enforcement have limited the impact of these measures; more broadly, low veterinary oversight of antimicrobial use further facilitates non-prescribed or inappropriate use in food animals (12). Similarly, in Bangladesh, antimicrobial sales often bypass prescriptions: farmers and feed sellers routinely sell antibiotics without veterinary approval, and access remains largely unregulated (14). Moreover, AMU in poultry farming has historically been driven not only by therapeutic needs but also by productivity, especially in low- and middle-income countries (LMICs) (15, 16).

In 2020, Indonesia implemented a nationwide ban on colistin use in livestock through a Ministry of Agriculture regulation, as part of broader national efforts to control AMR and aligned with Indonesia’s AMR National Action Plan (17). The goal of this regulation was to stop the use of colistin as both a growth promoter and therapeutic in poultry production in order to safeguard the efficacy of colistin and reduce the burden of AMR (6). While such policy interventions, particularly outright bans on specific antibiotics such as colistin, can be enforced through supply-side restrictions without requiring changes in farmer knowledge or behavior, stewardship approaches that rely on the responsible use of still-available antibiotics are more dependent on the attitudes and practices of farmers and farm personnel. In these contexts, key stakeholders may lack sufficient training or may not be well-informed about the risks of AMR, which can become a barrier to the successful implementation of antibiotic stewardship programs (18–20). For instance, in Rwanda, inappropriate practices such as obtaining antibiotics from friends and neighbors, keeping leftover antibiotics for future use, and self-prescribing persist despite existing veterinary oversight regulations (21, 22).

Bans on antibiotics like colistin can reshape AMU patterns in livestock systems as restricting specific antibiotics triggers shifts toward alternative drug classes as farmers seek to maintain productivity or manage diseases once controlled by the prohibited compound (23). For example, it has been suggested that phasing out growth promoters in swine could be associated with increased usage levels of therapeutic antimicrobials and AMR among foodborne pathogens (10). This trend is amplified in transitioning economies like Indonesia, where rapid intensification of poultry production inherently relies on routine antimicrobial use, that is projected to surge by 202% by 2030 (24). Nevertheless, empirical data on how Indonesian farmers specifically adapted to the 2020 colistin ban, including impacts on disease management, productivity, or resistance outcomes, remain critically scarce. Indeed, low KAP levels and limited awareness of the ban could, in theory, obscure the intended impact of regulatory interventions, particularly if the ban primarily reduces use of the targeted antimicrobial while farmers compensate by substituting alternative antimicrobial molecules rather than decreasing overall antimicrobial reliance. Therefore, our goal was to investigate KAP levels of layer poultry farmers in Indonesia before the colistin ban, descriptively compare reported AMU patterns before and after the ban, and investigate sociodemographic characteristics associated with awareness of the colistin ban.

## Materials and methods

### Overview

We conducted two related but non-identical cross-sectional studies, one in 2014 and another in 2023, as part of the COINCIDE study (6). The 2014 pre-ban study aimed to assess the knowledge, attitudes, and practices (KAP) of layer poultry farmers regarding antimicrobial use (AMU) and antimicrobial resistance (AMR), whereas the 2023 post-ban study assessed farmers’ awareness of the colistin ban and their current AMU practices. Because the two studies had related but distinct objectives, they differed in questionnaire scope, sampling approach, and participant composition, and were not intended to represent a longitudinal follow-up of the same farms. Ethical approval for the 2014 pre-ban study was obtained with ethical certificate number LB.02.01/5.2/KE.011/2014 from the Health Research and Development Center of the Ministry of Health, and it was part of the study conducted between 2013 and 2016. Ethical approval for the 2023 study was obtained from the Faculty of Medicine, Universitas Indonesia—Cipto Mangunkusumo Hospital, Indonesia (KET-294/UN2. F1/ETIK/PPM.00.02/2022) and the National Research and Innovation Agency (BRIN), Indonesia (B-2109/II/HM.00.01/4/2022). Both phases were conducted in the same study location in Central Java, Indonesia: Sukoharjo, Karanganyar, and Klaten, and focused on small-scale layer farms, defined as having fewer than 11,500 layer chickens, because layer production involves long production cycles in which repeated AMU decisions may occur (Figure 1). Interviews and surveys were conducted after obtaining participants’ explicit, free, and informed prior consent (Table 1).

**Figure 1:**
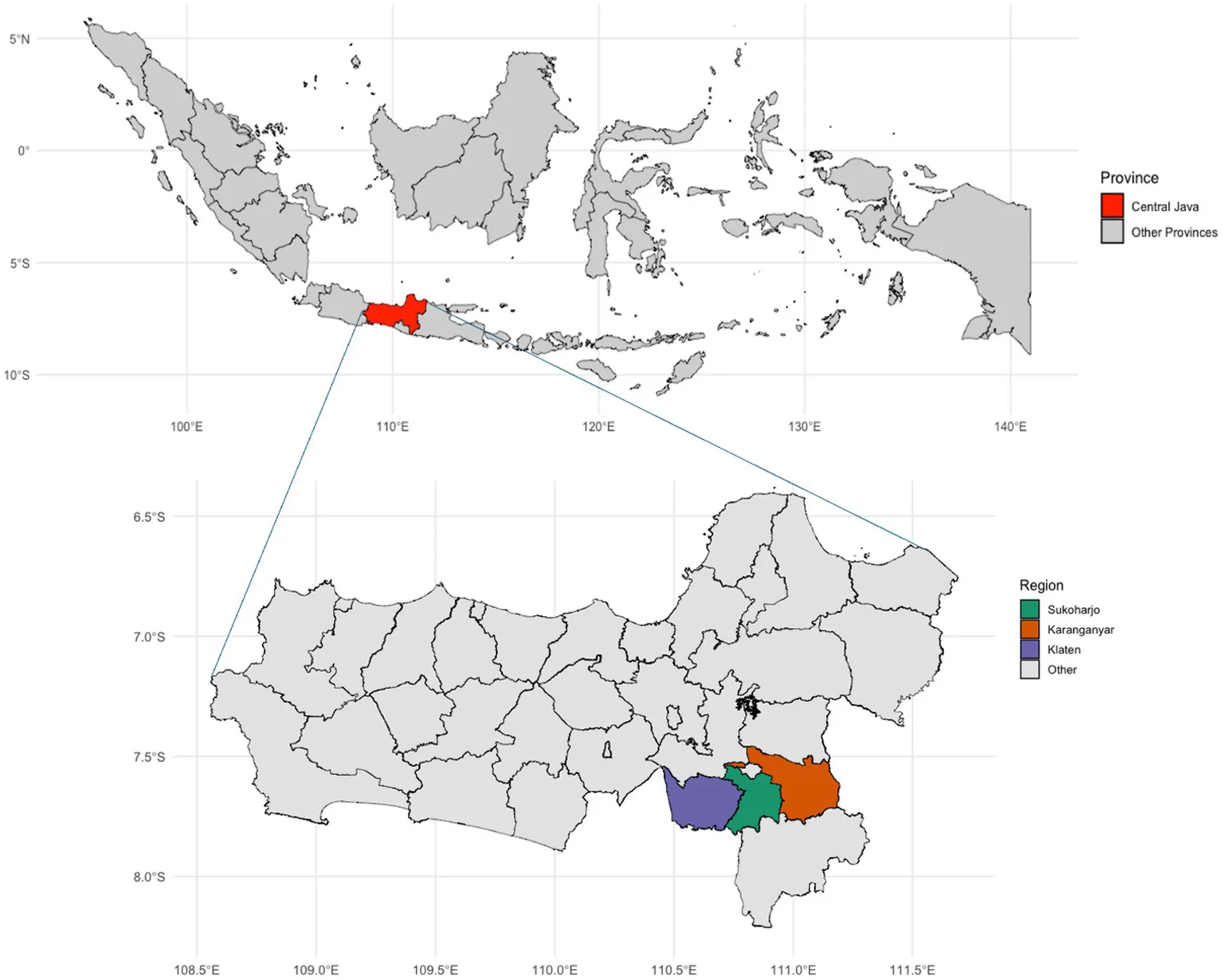
Geographical map of Central Java, Indonesia, highlighting the three regencies (Sukoharjo, Karanganyar, and Klaten) where the two studies were conducted.

**Table 1:** Distribution of high, moderate, and low scores regarding knowledge, attitude, and practice on antimicrobial use and resistance among poultry farmers in Indonesia before the colistin ban.

Category	High score (%)	Moderate score	Low score (%)
Knowledge^*^	11 (27.5%)	22 (55%)	7 (17.5%)
Attitude^*^	15 (37.5%)	17 (42.5%)	8 (20%)
Practice^**^	3 (7.5%)	25 (62.7%)	12 (30%)

^*^The Knowledge and Attitude section included 17 questions, with high scores defined as 66–100% (12–17), moderate as 31–65% (6–11), and low as ≤30% (0–5).

^**^The Practice section included 16 questions, with high scores defined as 66–100% (11–16), moderate as 31–65% (6–10), and low as ≤30% (0–5).

### First study: before the ban (2014)

The first study assessed the KAP of poultry farmers actively engaged in small-scale poultry production and documented the classes of antimicrobials they used. A cross-sectional survey was conducted among 40 small-scale layer poultry farmers. The sample size was determined based on the original COINCIDE study design and field feasibility. Farmers were randomly selected using a Probability Proportional to Size (PPS) sampling approach based on farm lists obtained from the local livestock office. The questionnaire contained questions spread across four sections: (1) demographics, (2) knowledge of AMU and AMR, (3) attitudes toward prudent AMU and AMR, and (4) practices related to AMU. The questionnaire was drafted by the Center for Indonesian Veterinary Analytical Studies (CIVAS) research team and internally reviewed by subject-matter experts within CIVAS. It was subsequently pre-tested in the community in Central Java to assess clarity and relevance of the questions, and the instrument was refined based on feedback obtained from this pilot exercise. Demographic data collected included gender, age (in years), role in animal husbandry, years of farming experience, educational background, and farm registration status. The knowledge and attitude sections contained 17 close-ended questions, while the practices section included 16 close-ended questions. Data were collected through structured questionnaire-based interviews using a questionnaire originally developed in Bahasa Indonesia (the translated version of the questions is available in Supplementary Tables 1–6).

### Second study: after the ban (2023)

The second study was conducted in 2023 to assess poultry farmers’ awareness of the national colistin ban, and antimicrobial classes currently being used. A Open Data Kit (ODK) digital survey consisting of six close-ended questions was used (25). The survey targeted individuals directly involved in poultry production and/or decision-making processes related to AMU and was conducted between October 29 and November 10, 2023. Small-scale layer farms with flock sizes ranging from 1,000 to 11,500 chickens were eligible for inclusion. The exact farms from the pre-ban study could not be included, as some farms no longer existed and contact information had been lost. Therefore, the same districts were selected for post-ban sampling to capture the same general target population of small-scale layer farms, rather than to conduct a farm-level longitudinal follow-up. In addition, eligibility criteria were provided to the District Animal Health Offices, which compiled lists of all potential farms in each district. From these lists, farms were initially selected using simple random sampling; however, because of recruitment constraints related to human sampling and animal boot swab sampling, the final selection was based on convenience sampling. The target sample size was 60 farms, with 20 farms selected from each district, based on the COINCIDE study protocol and field feasibility (6). In total, 60 farms were approached and included in the study, and 60 respondents were interviewed. The questionnaire, designed in Bahasa Indonesia, was structured into two sections: (1) demographics, and (2) awareness and practices related to AMU, AMR, and colistin use (the translated version of the questions is available in Supplementary Table 2). Respondents were asked to identify who is responsible for AMU on their farms, whether antimicrobials are currently used, and if so, which ones during specific farming periods. Additionally, the questionnaire inquired about the origin of antimicrobials, factors influencing AMU, farmers’ awareness of the colistin ban, and whether they use colistin on their farms, along with the reasons for its use or non-use (Table 2; Supplementary Tables 7–9).

**Table 2:** Distribution of responses regarding awareness of antimicrobial resistance and the colistin ban among poultry farmers after the implementation of the colistin ban.

Question	Response	Number of responses (%)
Who is responsible for deciding or permitting the use of drugs, especially antibiotics on your farm?	Farmworker	2 (3.6%)
	Other	6 (10.9%)
	Owner	43 (78.2%)
	Veterinarian	4 (7.3%)
Do you currently use antibiotics on your farm? If yes, record antibiotics used during pullet and laying periods.	No	8 (14.5%)
	Yes	47 (85.5%)
Where do the drugs, especially antibiotics used on your farm, come from?	With veterinary consultation	4 (7.3%)
	Farmworker	1 (1.8%)
	Other	5 (9.1%)
	Owner	45 (81.8%)
Based on whose consideration is the use of antibiotics in livestock carried out?	Drug-feed technical service	20 (36.4%)
	Lesson learnt	7 (12.7%)
	Other	19 (34.5%)
	Veterinarian	9 (16.4%)
Are you aware of the colistin ban in animals in Indonesia?	No	45 (81.8%)
	Yes	10 (18.2%)
Do you use colistin in your layer farm? If Yes, please specify the reason. If No, please specify the reason	No	55 (100%)

### Data analysis

Responses collected from in-person interviews were entered into a spreadsheet using Microsoft Excel, and the data were double-checked for completeness and accuracy. Data cleaning and analysis were performed using R (version 4.4.3). The assessment of KAP in the pre-ban study was conducted using a scoring system, where each participant’s score was calculated as the sum of correct responses (26). Correct answers were scored as 1, while incorrect answers were scored as 0. The correct responses were determined through expert consultation, including input from MP, DN, SYN, and AR, to ensure content validity. The questionnaire also included a “Do not know” option for most questions, which was conservatively scored as 0 (e.g., incorrect), as this response was interpreted as indicative of a lack of knowledge. The total scores for knowledge, attitude, and practice were obtained by summing the correct or appropriate responses within each domain, with maximum possible scores of 17 for knowledge, 17 for attitude, and 16 for practice. Scores were then categorized into three levels: High (66–100% of the maximum score: 12–17 for knowledge and attitude, 11–16 for practice), Moderate (31–65%: 6–11 for knowledge and attitude, 6–10 for practice), and Low (≤30%: 0–5 for knowledge, attitude, and practice) (27–29). To assess the relationship between the responses to each question (correct vs. incorrect) and categorical predictors such as gender, education level, and farm registration status, we performed Fisher’s Exact Test. For continuous predictors such as age and years of farming experience, we applied the Kruskal-Wallis test to examine differences in their distributions based on the responses. In both the pre-ban and post-ban questionnaires, farmers were asked to report the names of antimicrobials used on their farms. Although some antimicrobials were used either individually or in combination, the reported names were subsequently categorized into their corresponding classes to facilitate the analysis of overall AMU patterns (Supplementary Figures 1, 2). To explore differences in the reported distribution of antimicrobial classes between the two periods, Fisher’s Exact Test was employed. Given the differences in sampling strategy, questionnaire structure, and study participants between the pre-ban and post-ban surveys, these comparisons were interpreted as descriptive and exploratory rather than as causal estimates of the effect of the colistin ban. For the post-ban data, the association between responses to each question and demographic variables was assessed using Fisher’s Exact Test or Kruskal-Wallis. The significance level for all statistical tests was set at 0.05.

## Results

Before the colistin ban, 27.5% (*n* = 11) of poultry farmers demonstrated a high score of knowledge regarding AMR and AMU, while the majority were classified as having moderate (55%, *n* = 22) or low (17.5%, *n* = 7) knowledge. In terms of attitude, 37.5% (*n* = 15) of participants exhibited a high score, while 42.5% (*n* = 17) scored moderately and 20% (*n* = 8) scored low. The lowest scores were observed in practice-related questions, with only 7.5% (*n* = 3) of participants classified as having good practices, whereas the majority fell into moderate (62.5%, *n* = 25) and low (30%, *n* = 12) categories (Table 1). There was no statistically significant association between demographic factors and KAP scores. Detailed responses to each question are presented in Supplementary Tables 1–6.

Before the ban, the most commonly antimicrobial classes in use as reported by farmers were tetracyclines (22%) and fluoroquinolones (18%). Other frequently reported classes included macrolides (14.7%), aminoglycosides (13.3%), and beta-lactams (10.7%). Colistin was used on 4.7% of farms (Figure 2). Following the colistin ban, tetracyclines, fluoroquinolones, and aminoglycosides were reported to be used by 21.7, 13, and 12% of farmers, respectively, which was not statistically different from pre-ban. No farm reported the use of colistin after the ban. Likewise, the proportion of farms reporting the use of macrolides, sulfonamides, and lincosamides were 15.2, 14.1, and 5.4%, respectively, which was not statistically different (Figure 2).

**Figure 2:**
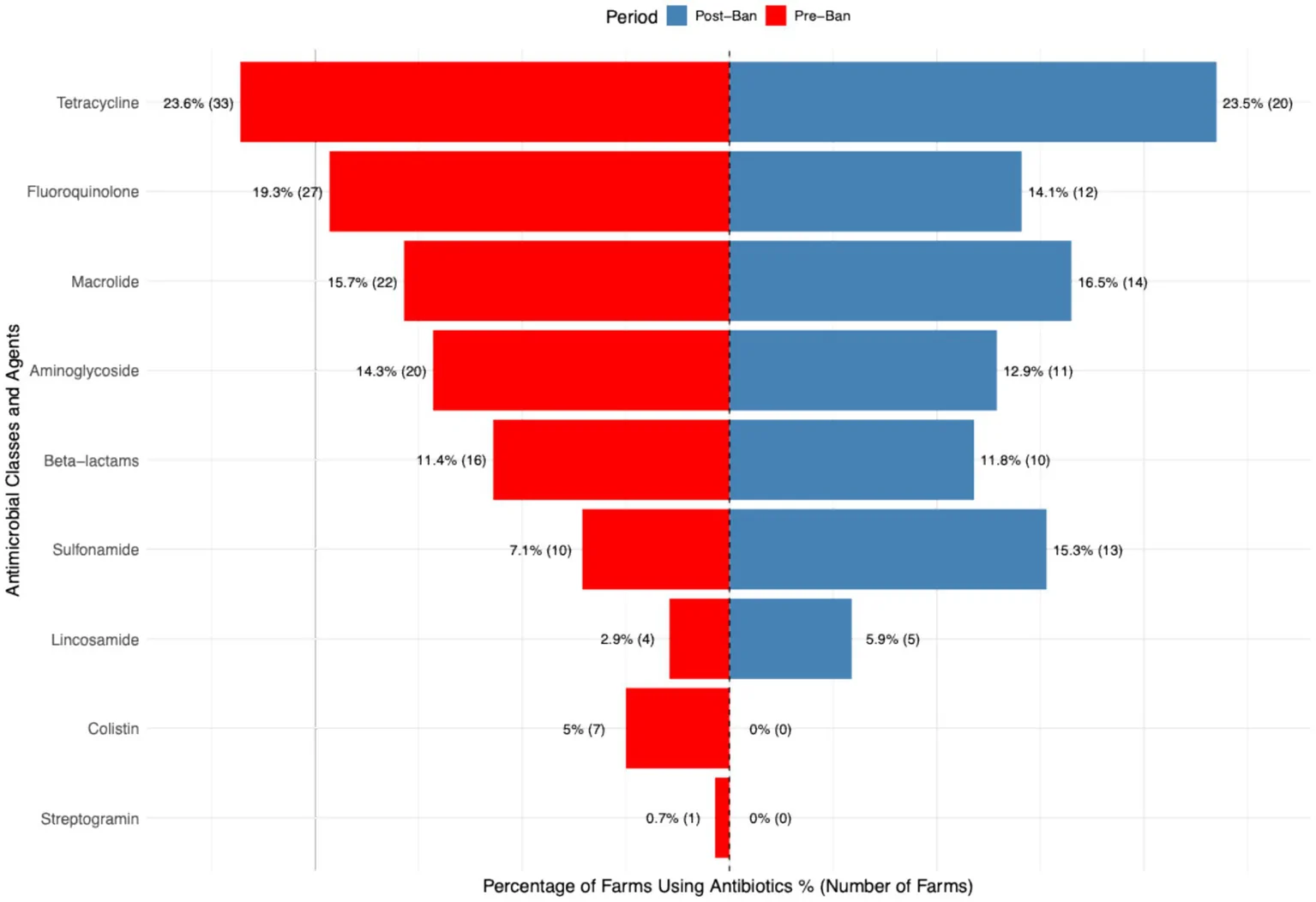
Comparison of antibiotic class usage reported by layer poultry farms in Indonesia before and after the colistin ban.

The post ban study demonstrated that 85.5% of respondents reported that antimicrobials were used on their farms; among those, the majority were male (77.4%), and with pre-college education (no formal schooling, elementary, junior high, or senior high school; Table 2; Supplementary Tables 7, 8). When asked about awareness of the colistin ban in animals in Indonesia, only 18.2% of respondents reported being aware of the ban. Among those who were aware, 90% were male. This group had a mean age of 41.6 years (±12) and an average of farm experience that was not statistically different from those unaware. In terms of education, 20% had tertiary education, and 80% had secondary or primary education. Additionally, 20% of respondents in the aware group owned registered farms. Among those unaware of the ban, 89.1% were male. Their mean age was 46.5 years (±14), and they had comparable farm experience. Regarding education, 17.8% had tertiary education, and the majority (82.2%) had secondary or primary education. There were no statistically significant differences in awareness by gender, education level, age, or farm registration status, as shown by logistic regression results (all *p* > 0.05; Table 3).

**Table 3:** Responses to the question “Are you aware of the colistin ban in animals in Indonesia?” by demographic variables.

Variables		Ban awareness		Odds Ratio (95% CL)	*p*-value
		Yes	No		
Gender	Male	9 (18%)	41 (82%)	0.8 (00.1–16.99)	0.851
	Female	1 (20%)	4 (80%)		
Education level^*^	Tertiary	2 (20%)	8 (80%)	2.83 (0.17–8.29)	0.478
	Secondary	7 (18.9%)	30 (81.1%)	2.7 (0.28–6.44)	0.440
	Primary	1 (12.5%)	7 (87.5%)		
Farm registration	Register	3 (20%)	12 (80%)	1.19 (0.21–5.77)	0.83
	Not-register	7 (17.5%)	33 (82.5%)		
Age		41.6 (±12)	46.5 (±14)	0.96 (0.88–1.02)	0.267
Farm experience (years)		12.6 (±11.5)	13.2 (±10.7)	1.03 (0.94–1.13)	0.569

^*^Education levels were categorized as follows: Primary included respondents with elementary education or no formal schooling (noschool); Secondary included those who completed junior high or senior high school; and Tertiary included individuals with a diploma, bachelor’s degree (diplomabachelor), or a master’s degree.

None of the respondents reported using the colistin after the ban. The majority of respondents (78.2%) reported that the owner was responsible for deciding or permitting the use of drugs, especially antimicrobials, on their farms (Table 2; Supplementary Table 2). Among owner decision-makers, most were male (91.4%), an average of 14.5 years of farm experience, and only 29% had registered farms. When asked whose consideration influenced AMU, veterinarians were cited most often, with respondents in this group generally younger (mean age 39), more educated, and associated with more registered farms (62.5%); however, these differences were not statistically significant (*p* > 0.05; Table 2; Supplementary Tables 7–9).

## Discussion

This study investigates the KAP of layer poultry farmers in Indonesia regarding AMU and AMR prior to the national colistin ban, as well as their awareness and use of colistin following the ban’s implementation. Given the rising global concern surrounding AMU and AMR over the past two decades and the important role farmers play in shaping antimicrobial practices (30–32), we assessed their knowledge and behaviors, which is essential for designing future targeted interventions to promote responsible AMU. Aligned with the One Health approach, such efforts will be essential to effectively combat AMR and to ensure the long-term health, productivity, and sustainability of poultry production systems, particularly in low-resource settings (33). It is important to note that this study focused exclusively on small-scale layer farms in Central Java, which, while a significant component of the Indonesian poultry sector, may not fully represent larger or integrated operations. Small-scale farms may operate with limited resources and distinct management practices, which may influence AMU patterns and AMR awareness. In addition, small-scale farms may lack consistent access to veterinary services or on-farm attending veterinarians, leading to self-diagnosis, over-the-counter antimicrobial purchases, and treatment without formal oversight (31, 34, 35).

The pre-ban assessment KAP among poultry farmers in Indonesia revealed generally moderate and low levels of awareness and engagement regarding AMR. The finding that only 27.5% of layer farmers demonstrated good knowledge aligns with those from studies carried out in other regions that have also reported limited understanding of AMR among poultry farmers. In India, 80% of poultry farmers in Telangana failed to follow proper dosage or withdrawal periods and frequently purchased antimicrobials over the counter; in Bangladesh, most farmers demonstrated insufficient knowledge, attitudes, and practices often relying on non-veterinary sources, with education and experience influencing KAP levels; and in Cameroon, rural farmers showed poor knowledge and practices, with improved biosecurity and higher education levels associated with better AMU behavior and risk perception (32, 36, 37).

Targeted training and educational programs are needed to ensure that, in addition to raising farmers’ knowledge and attitudes, their practices are also improved. This highlights the importance of bridging the gap between awareness and actual behavior change (26). Indeed, farmers’ low knowledge scores may be linked to a lack of formal training in poultry farming and limited consultation with animal health professionals (32). Since educational status is important in combating AMR, training can help establish a strong foundation in AMU, hygiene, and biosecurity (38, 39).

The implications of low baseline KAP levels are significant. Inappropriate AMU practices, driven by inadequate KAP, contribute to the development and spread of AMR, posing a threat to both animal and human health (40). Interestingly, we did not find a statistically significant association between demographic factors and KAP scores, suggesting that inadequate KAP was equally disseminated in all population strata. Conversely, demographic and socioeconomic variables, such as gender and education level, were significantly associated with KAP in Nepal and Bangladesh, which suggest that poultry farming industries in different regions may adopt different strategies to increase KAP levels (26, 36).

We explored changes in AMU patterns among poultry farmers in Indonesia following the colistin ban. Prior to the ban, tetracyclines, fluoroquinolones, and aminoglycosides were the most frequently utilized antimicrobial classes. Similar patterns of AMU have been reported in various LMICs, where the use of broad-spectrum antimicrobials is widespread in poultry production systems (41, 42). In Nepal and Pakistan, the use of tetracyclines, particularly doxycycline, as well as macrolides, aminoglycosides, and polymyxins such as colistin, have been highlighted (26, 43, 44). Long-term AMU monitoring in these regions has shown a continuous dependence on the mentioned antimicrobials in poultry farming, raising concerns about the consequences for AMR in both animal and human health (36, 44, 45). In our study, changes in the use of these antimicrobial classes post-ban were not statistically significant which alleviates concerns about potential antimicrobial replacement. This phenomenon, where the restriction of one antimicrobial leads to increased use of others, has been observed in other contexts and may contribute to the selection of AMR to these alternative antimicrobials (46–48). Respondents reported no colistin use after the ban, but low awareness suggests this pattern may be driven by limited availability (e.g., supply restrictions) rather than awareness-based behavior change. We also did not assess whether reduced access affected disease occurrence, productivity, or animal welfare.

The post-ban study reveals that a high percentage of farmers (85.5%) reported using antimicrobials on their farms, indicating that antimicrobials remain commonly used in poultry production after the colistin ban in Indonesia. A 2023 study conducted post-colistin ban on broiler farms in West Java supports these findings, revealing widespread use of antimicrobials such as fluoroquinolones, macrolides, tetracyclines, colistin, and penicillins, primarily for preventive purposes. The West Java study also highlighted the frequent preventive use of Highest Priority Critically Important Antimicrobials (HPCIAs), underscoring the urgent need for targeted interventions and stricter stewardship policies to promote prudent AMU in Indonesian poultry production (49). Our study revealed that the majority of antimicrobial-using respondents were male (77.4%), consistent with previous work done in the region (26, 45). This is likely due to the higher number of male poultry farmers compared to female farmers in Indonesia (15, 50).

Our survey revealed that 18.2% of respondents were aware of the colistin ban, suggesting a significant gap in the dissemination of information and regulations to poultry farmers. This lack of awareness may be linked to the same under arching construct that resulted in low levels of KAP among poultry farmers prior to the ban, which represents a critical concern, as it can hinder the effectiveness of future interventions aimed at reducing AMR, and undermine future industry-led initiatives. The finding that veterinarians are frequently cited as influencing AMU, particularly among younger and more educated farmers with registered farms, highlights the significance of veterinary guidance in promoting responsible AMU, which provides an avenue to be explored to increase KAP in AMU and resistance among poultry farmers in Indonesia. The importance of veterinarians’ opinions and advice regarding AMU has been frequently described (40, 51, 52).

### Strengths and limitations

This study examined the KAP of small-scale layer poultry farmers regarding AMR and AMU, both before and after the national colistin ban. It identifies gaps in AMR-related practices and informs policymaking using the KAP framework. The following limitations should be considered when interpreting these findings. As described in the Methods, the pre- and post-ban surveys differed in scope, questionnaire content, sampling approach, and participant composition. The pre-ban phase used a comprehensive KAP questionnaire, whereas the post-ban phase employed a shorter instrument focused primarily on awareness and current practices. Therefore, comparisons between the two periods should be interpreted as descriptive and exploratory rather than as direct longitudinal evidence of policy impact. Second, participant selection differed between the two periods. The pre-ban survey used PPS-based sampling, while the post-ban survey relied on convenience sampling due to recruitment constraints, and the relatively modest sample sizes may have reduced statistical power. Third, the study included only small-scale layer farms (<11,500 birds) in three districts, which limits the generalizability of the findings to larger farms, other poultry production sectors, or other regions. Fourth, antimicrobial use (AMU) data were primarily self-reported and only partially corroborated; there was no direct verification of post-ban colistin-use statements, which may be subject to underreporting following the ban. Finally, the analyses were largely bivariate and exploratory, and unmeasured confounding cannot be ruled out.

## Conclusion

Here, we compared layer poultry farmers’ KAP and awareness regarding AMU and AMR in Indonesia before and after the national colistin ban. The results from the pre-ban study revealed limited KAP related to AMU and AMR. Although colistin itself no longer appeared to be in use, the colistin ban did not result in substantial shifts in overall AMU patterns. We also identified a significant gap in farmers’ awareness of the colistin ban, with only 18.2% of farmers reporting awareness the ban, and no significant association observed with any investigated demographic factors. These findings reinforce the need for effective communication strategies, including educational programs that actively involve veterinarians, to ensure regulatory changes are properly disseminated to all poultry farmers.

## Data Availability

The raw data supporting the conclusions of this article will be made available by the authors, without undue reservation.

## References

[1] Chapot L Hibbard R Ariyanto KB Maulana KY Yusuf H Febriyani W . Needs and capabilities for improving poultry production and health management in Indonesia. PLoS One. (2024) 19:e0308379. doi: 10.1371/journal.pone.0308379, 39172873 PMC11340977

[2] Coyne L Arief R Benigno C Giang VN Huong LQ Jeamsripong S . Characterizing antimicrobial use in the livestock sector in three south east Asian countries (Indonesia, Thailand, and Vietnam). Antibiotics. (2019) 8:33. doi: 10.3390/antibiotics8010033, 30934638 PMC6466601

[3] Wibisono F Sumiarto B Untari T Effendi M Permatasari D Witaningrum A. Antimicrobial resistance on *Escherichia coli* from poultry production on Blitar, Indonesia. Indian J Forensic Med Toxicol. (2020) 14:4131–6. doi: 10.37506/ijfmt.v14i4.12288

[4] Morrow CJ. Antimicrobial resistance (AMR): an important one health issue for layer and meat poultry industries worldwide. Poult Sci. (2024) 103:103690. doi: 10.1016/j.psj.2024.103690, 38663076 PMC11063636

[5] Mondal AH Khare K Saxena P Debnath P Mukhopadhyay K Yadav D. A review on colistin resistance: an antibiotic of last resort. Microorganisms. (2024) 12:772. doi: 10.3390/microorganisms12040772, 38674716 PMC11051878

[6] Naing SY Severin J Zomer A Kuntaman K Suandy I Sunandar S . Impact of reducing colistin use on colistin resistance in *Escherichia coli* isolated from humans and poultry in Indonesia (COINCIDE): a protocol for a multisectoral, transdisciplinary one health study. One Health. (2026) 22:101347. doi: 10.1016/j.onehlt.2026.101347, 41704561 PMC12907006

[7] Lemlem M Aklilu E Mohamed M Kamaruzzaman NF Zakaria Z Harun A . Phenotypic and genotypic characterization of colistin-resistant *Escherichia coli* with mcr-4, mcr-5, mcr-6, and mcr-9 genes from broiler chicken and farm environment. BMC Microbiol. (2023) 23:392. doi: 10.1186/s12866-023-03118-y, 38062398 PMC10704802

[8] Sohail MN Isloor S Rathnamma D Chandra Priya S Veeregowda BM Hegde NR . Phenotypic and genotypic characterization of colistin, ESBL, and multidrug resistance in *Escherichia coli* across the broiler production chain in Karnataka, India. Poultry. (2025) 4:51. doi: 10.3390/poultry4040051

[9] Chilanga F Kasozi KI Mazeri S Paterson GK Muwonge A. A systematic review of antimicrobial resistance transmission inferences at the human-livestock interface in Africa. npj Antimicrob Resist. (2025) 3:58. doi: 10.1038/s44259-025-00126-y, 40588479 PMC12209416

[10] Wen R Li C Zhao M Wang H Tang Y. Withdrawal of antibiotic growth promoters in China and its impact on the foodborne pathogen *Campylobacter coli* of swine origin. Front Microbiol. (2022) 13:1004725. doi: 10.3389/fmicb.2022.1004725, 36160189 PMC9495300

[11] Dimuccio MM Conforti V Celentano FE Circella E Salvaggiulo A Bozzo G . Regulation of antibiotic use in livestock: European and international strategies to prevent and control antimicrobial resistance and ensure animal welfare. Antibiotics (Basel). (2026) 15:67. doi: 10.3390/antibiotics15010067, 41594105 PMC12837735

[12] Lambrou AS Innes GK O’Sullivan L Luitel H Bhattarai RK Basnet HB . Policy implications for awareness gaps in antimicrobial resistance (AMR) and antimicrobial use among commercial Nepalese poultry producers. Glob Health Res Policy. (2021) 6:6. doi: 10.1186/s41256-021-00187-2, 33546762 PMC7866638

[13] Sani AA Rafiq K Akter F Islam P Sachi S Sultana N . Effect of knowledge of informal poultry drug prescribers on their attitude and practice toward antimicrobial use, residues, and resistance in Bangladesh. Vet World. (2023) 16:1821–8. doi: 10.14202/vetworld.2023.1821-1828, 37859963 PMC10583889

[14] Kalam MA Alim MA Shano S Nayem MRK Badsha MR Mamun MAA . Knowledge, attitude, and practices on antimicrobial use and antimicrobial resistance among poultry drug and feed sellers in Bangladesh. Vet Sci. (2021) 8:111. doi: 10.3390/vetsci8060111, 34203812 PMC8232782

[15] Coyne L Patrick I Arief R Benigno C Kalpravidh W McGrane J . The costs, benefits and human behaviours for antimicrobial use in small commercial broiler chicken systems in Indonesia. Antibiotics. (2020) 9:154. doi: 10.3390/antibiotics9040154, 32244693 PMC7235826

[16] khoubfekr H Abdollahi M Jokar M Amiri H Babazadeh S Rahmanian V . Risk factors for coronavirus disease 2019 (COVID-19)-related mortality among hospitalized patients in southeastern Iran: a case-control study. J Adv Biomed Sci. (2024) 14:155–166. doi: 10.18502/jabs.v14i2.15752

[17] The Ministry of Agriculture of the Republic of Indonesia (Ed.). National Action Plan on Antimicrobial Resistance in Indonesia 2020–2024 Jakarta, Indonesia: (2022).

[18] Kakkar AK Shafiq N Singh G Ray P Gautam V Agarwal R . Antimicrobial stewardship programs in resource constrained environments: understanding and addressing the need of the systems. Front Public Health. (2020) 8:00140. doi: 10.3389/fpubh.2020.00140 PMC7198767 32411647

[19] Alghamdi S Atef-Shebl N Aslanpour Z Berrou I. Barriers to implementing antimicrobial stewardship programmes in three Saudi hospitals: evidence from a qualitative study. J Glob Antimicrob Resist. (2019) 18:284–90. doi: 10.1016/j.jgar.2019.01.031, 30797087 PMC7104169

[20] McKernan C Benson T Farrell S Dean M. Antimicrobial use in agriculture: critical review of the factors influencing behaviour. JAC Antimicrob Resist. (2021) 3:dlab178. doi: 10.1093/jacamr/dlab178, 34859222 PMC8634307

[21] Caudell MA Kiambi S Afakye K Koka E Kabali E Kimani T . Social-technical interventions to reduce antimicrobial resistance in agriculture: evidence from poultry farmer field schools in Ghana and Kenya. JAC-Antimicrob Resist. (2022) 4:dlab193. doi: 10.1093/jacamr/dlab193, 35156026 PMC8826779

[22] Hirwa EM Mujawamariya G Shimelash N Shyaka A. Evaluation of cattle farmers’ knowledge, attitudes, and practices regarding antimicrobial use and antimicrobial resistance in Rwanda. PLoS One. (2024) 19:e0300742. doi: 10.1371/journal.pone.0300742, 38603685 PMC11008905

[23] Tang KL Caffrey NP Nóbrega DB Cork SC Ronksley PE Barkema HW . Restricting the use of antibiotics in food-producing animals and its associations with antibiotic resistance in food-producing animals and human beings: a systematic review and meta-analysis. Lancet Planet Heath. (2017) 1:e316–27. doi: 10.1016/S2542-5196(17)30141-9, 29387833 PMC5785333

[24] Van Boeckel TP Brower C Gilbert M Grenfell BT Levin SA Robinson TP . Global trends in antimicrobial use in food animals. Proc Natl Acad Sci. (2015) 112:5649–54. doi: 10.1073/pnas.1503141112, 25792457 PMC4426470

[25] Hartung C. Lerer A. Anokwa Y. Tseng C. Brunette W. Borriello G. Open data kit: tools to build information services for developing regions. Proceedings of the 4th ACM/IEEE International Conference on Information and Communication Technologies and Development. ICTD’10. New York Association for Computing Machinery (2010). p. 1–12

[26] Subedi D Jyoti S Thapa B Paudel S Shrestha P Sapkota D . Knowledge, attitude, and practice of antibiotic use and resistance among poultry farmers in Nepal. Antibiotics. (2023) 12:1369. doi: 10.3390/antibiotics12091369, 37760666 PMC10525118

[27] Andrade C Menon V Ameen S Kumar Praharaj S. Designing and conducting knowledge, attitude, and practice surveys in psychiatry: practical guidance. Indian J Psychol Med. (2020) 42:478–81. doi: 10.1177/0253717620946111, 33414597 PMC7750837

[28] Precha N Sukmai S Hengbaru M Chekoh M Laohaprapanon S Makkaew P . Knowledge, attitudes, and practices regarding antibiotic use and resistance among health science and non-health science university students in Thailand. PLoS One. (2024) 19:e0296822. doi: 10.1371/journal.pone.0296822, 38180959 PMC10769033

[29] Wang L Abualfoul M Oduor H Acharya P Cui M Murray A . A cross-sectional study of knowledge, attitude, and practice toward COVID-19 in solid organ transplant recipients at a transplant center in the United States. Front Public Health. (2022) 10:880774. doi: 10.3389/fpubh.2022.880774, 36211649 PMC9539443

[30] Hoque R Ahmed SM Naher N Islam MA Rousham EK Islam BZ . Tackling antimicrobial resistance in Bangladesh: a scoping review of policy and practice in human, animal and environment sectors. PLoS One. (2020) 15:e0227947. doi: 10.1371/journal.pone.0227947, 31986167 PMC6984725

[31] Nhung NT Chansiripornchai N Carrique-Mas JJ. Antimicrobial resistance in bacterial poultry pathogens: a review. Front Vet Sci. (2017) 4:126. doi: 10.3389/fvets.2017.00126, 28848739 PMC5554362

[32] Moffo F Mouliom Mouiche MM Kochivi FL Dongmo JB Djomgang HK Tombe P . Knowledge, attitudes, practices and risk perception of rural poultry farmers in Cameroon to antimicrobial use and resistance. Prev Vet Med. (2020) 182:105087. doi: 10.1016/j.prevetmed.2020.105087, 32726706

[33] Singh S Kriti M Anamika KS Sharma P Pal N Sarma DK . A one health approach addressing poultry-associated antimicrobial resistance: human, animal and environmental perspectives. Microbe. (2025) 7:100309. doi: 10.1016/j.microb.2025.100309

[34] Graham JP Eisenberg JNS Trueba G Zhang L Johnson TJ. Small-scale food animal production and antimicrobial resistance: mountain, molehill, or something in-between? Environ Health Perspect. (2017) 125:104501. doi: 10.1289/EHP2116, 29038091 PMC5933306

[35] Gemeda BA Amenu K Magnusson U Dohoo I Hallenberg GS Alemayehu G . Antimicrobial use in extensive smallholder livestock farming systems in Ethiopia: knowledge, attitudes, and practices of livestock keepers. Front Vet Sci. (2020) 7:55. doi: 10.3389/fvets.2020.00055, 32175334 PMC7055293

[36] Hassan M. M. Kalam M. A. Alim M. A. Shano S. Nayem M. R. K. Badsha M. R. Knowledge, attitude, and practices on antimicrobial use and antimicrobial resistance among commercial poultry farmers in Bangladesh. Antibiotics (Basel) (2021) 10:784. doi: 10.3390/antibiotics10070784, 34203195 PMC8300693 34203195

[37] Ramesh N Tripathi H. Antibiotic usage practice and knowledge on antimicrobial resistance among livestock and poultry farmers of Telangana state, India. Indian J Anim Sci. (2022) 92:766–173. doi: 10.56093/ijans.v92i2.122074

[38] Harbarth S Balkhy HH Goossens H Jarlier V Kluytmans J Laxminarayan R . Antimicrobial resistance: one world, one fight! Antimicrob Resist Infect Control. (2015) 4:49. doi: 10.1186/s13756-015-0091-2

[39] Dhaka P Chantziaras I Vijay D Bedi JS Makovska I Biebaut E . Can improved farm biosecurity reduce the need for antimicrobials in food animals? A scoping review. Antibiotics (Basel). (2023) 12:893. doi: 10.3390/antibiotics12050893, 37237795 PMC10215210

[40] Borelli E Ellis K Tomlinson M Hotchkiss E. Antimicrobial usage and resistance in Scottish dairy herds: a survey of farmers’ knowledge, behaviours and attitudes. BMC Vet Res. (2023) 19:72. doi: 10.1186/s12917-023-03625-0, 37208702 PMC10197045

[41] Malik B Bhattacharyya S. Antibiotic drug-resistance as a complex system driven by socio-economic growth and antibiotic misuse. Sci Rep. (2019) 9:9788. doi: 10.1038/s41598-019-46078-y, 31278344 PMC6611849

[42] Ventola CL. The antibiotic resistance crisis: part 1: causes and threats. Pharm Ther. (2015) 40:277. PMC4378521 25859123

[43] Dhakal S Gompo TR. Assessment of farmer’s knowledge of antimicrobial resistance, their practice of antimicrobial usage and biosecurity status of poultry farms in Kathmandu Valley and Chitwan District, Nepal. Int J Appl Sci Biotechnol. (2022) 10:50–9. doi: 10.3126/ijasbt.v10i1.41675

[44] Umair M Tahir MF Ullah RW Ali J Siddique N Rasheed A . Quantification and trends of antimicrobial use in commercial broiler chicken production in Pakistan. Antibiotics (Basel). (2021) 10:598. doi: 10.3390/antibiotics10050598, 34069928 PMC8157606

[45] Shahi MK Gompo TR Sharma S Pokhrel B Manandhar S Jeamsripong S. Situational analysis and knowledge, attitudes, and practices of antimicrobial use and resistance among broiler poultry farmers in Nepal. Animals (Basel). (2023) 13:3135. doi: 10.3390/ani13193135, 37835741 PMC10571899

[46] Marshall DA McGeer A Gough J Grootendorst P Buitendyk M Simonyi S . Impact of antibiotic administrative restrictions on trends in antibiotic resistance. Can J Public Health. (2006) 97:126–31. doi: 10.1007/BF03405330, 16620000 PMC6975704

[47] Church NA McKillip JL. Antibiotic resistance crisis: challenges and imperatives. Biologia (Bratisl). (2021) 76:1535–50. doi: 10.1007/s11756-021-00697-x

[48] Pujol M Gudiol F. Evidence for antibiotic cycling in control of resistance. Curr Opin Infect Dis. (2001) 14:711–5. doi: 10.1097/00001432-200112000-00008, 11964889

[49] Anwar Sani R Sunandar S Rachmawati A Pertela G Susanti O Rahayu KP . Antimicrobial usage and antimicrobial resistance in commensal *Escherichia coli* from broiler farms: a farm-level analysis in West Java, Indonesia. Antibiotics (Basel). (2024) 13:1181. doi: 10.3390/antibiotics13121181, 39766571 PMC11672412

[50] Dentzman K Lewin P. A fair comparison: women’s and men’s farms at seven scales in the United States. Rural Sociol. (2024) 89:3–39. doi: 10.1111/ruso.12512

[51] Coyne LA Pinchbeck GL Williams NJ Smith RF Dawson S Pearson RB . Understanding antimicrobial use and prescribing behaviours by pig veterinary surgeons and farmers: a qualitative study. Vet Rec. (2014) 175:593–3. doi: 10.1136/vr.102686, 25200432

[52] Vijay D Bedi JS Dhaka P Singh R Singh J Arora AK . Knowledge, attitude, and practices (KAP) survey among veterinarians, and risk factors relating to antimicrobial use and treatment failure in dairy herds of India. Antibiotics. (2021) 10:216. doi: 10.3390/antibiotics10020216, 33671483 PMC7926553

