# Supplementary material for: Impact of the colistin ban on antimicrobial use and resistance-related practices among layer farmers in Indonesia: a comparative KAP study
Source: Front Vet Sci. 2026 Sep 23;13:1885994. doi: 10.3389/fvets.2026.1885994 (PMC13671022; doi:10.3389/fvets.2026.1885994)
Supplement: Supplementary file 1 [file Supplementary_file_1.DOCX]

**Appendix**

| **Table 1. Knowledge questions from the pre-ban questionnaire with correct (“Yes”) and incorrect (“No” or “Do not know”) answers, presented by gender and farm position.** | | | | | | | |
| --- | --- | --- | --- | --- | --- | --- | --- |
|  |  | **Gender** | | **Position in Farm** | | | |
| **Question** | **Answers** | **Male** | **Female** | **Manager** | **Owner** | **head of the cage** | **health expert** |
| Antibiotics are drugs that can be used to treat all kinds of diseases? | Correct Answer | 15(37.5%) | 3(7.5%) | 3(60%) | 11(36.67%) | 2(66.67%) | 2(100%) |
|  | Incorrect Answer | 18(45%) | 4(10%) | 2(40%) | 19(63.33%) | 1(33.33%) | 0(0%) |
| Disease treatment should use one type of antibiotic in the long term without having to rotate with other types? | Correct Answer | 20(50%) | 5(12.5%) | 4(80%) | 16(53.33%) | 3(100%) | 2(100%) |
|  | Incorrect Answer | 13(32.5%) | 2(5%) | 1(20%) | 14(46.67%) | 0(0%) | 0(0%) |
| The use of antibiotics for the treatment of disease should be consulted with competent medical personnel | Correct Answer | 23(57.5%) | 4(10%) | 4(80%) | 19(63.33%) | 2(66.67%) | 2(100%) |
|  | Incorrect Answer | 10(25%) | 3(7.5%) | 1(20%) | 11(36.67%) | 1(33.33%) | 0(0%) |
| Antibiotic compounds may not be traded freely (without a prescription/consultation of medical personnel)? | Correct Answer | 13(32.5%) | 1(2.5%) | 3(60%) | 8(26.67%) | 1(33.33%) | 2(100%) |
|  | Incorrect Answer | 20(50%) | 6(15%) | 2(40%) | 22(73.33%) | 2(66.67%) | 0(0%) |
| Antibiotic compounds contained in feed should not be of the same type as antibiotics used for treatment in humans? | Correct Answer | 19(47.5%) | 7(17.5%) | 3(60%) | 20(66.67%) | 3(100%) | 0(0%) |
|  | Incorrect Answer | 14(35%) | 0(0%) | 2(40%) | 10(33.33%) | 0(0%) | 2(100%) |
| The use of antibiotics for treatment should be determined based on the cause of the disease? | Correct Answer | 28(70%) | 5(12.5%) | 5(100%) | 23(76.67%) | 3(100%) | 2(100%) |
|  | Incorrect Answer | 5(12.5%) | 2(5%) | 0(0%) | 7(23.33%) | 0(0%) | 0(0%) |
| Use of antibiotics less than the prescribed dose for treatment can cause antibiotic resistance? | Correct Answer | 12(30%) | 1(2.5%) | 2(40%) | 8(26.67%) | 2(66.67%) | 1(50%) |
|  | Incorrect Answer | 21(52.5%) | 6(15%) | 3(60%) | 22(73.33%) | 1(33.33%) | 1(50%) |
| Can the continuous use of antibiotics lead to antibiotic resistance? | Correct Answer | 21(52.5%) | 5(12.5%) | 5(100%) | 16(53.33%) | 3(100%) | 2(100%) |
|  | Incorrect Answer | 12(30%) | 2(5%) | 0(0%) | 14(46.67%) | 0(0%) | 0(0%) |

| **Table 1. Continued** |  |  |  |  |  |  |  |
| --- | --- | --- | --- | --- | --- | --- | --- |
| Antibiotics in their use must follow the provisions of the drug stop time? | Correct Answer | 23(57.5%) | 4(10%) | 4(80%) | 20(66.67%) | 2(66.67%) | 1(50%) |
|  | Incorrect Answer | 10(25%) | 3(7.5%) | 1(20%) | 10(33.33%) | 1(33.33%) | 1(50%) |
| Uncontrolled use of antibiotics can lead to antibiotic resistance? | Correct Answer | 19(47.5%) | 3(7.5%) | 4(80%) | 13(43.33%) | 3(100%) | 2(100%) |
|  | Incorrect Answer | 14(35%) | 4(10%) | 1(20%) | 17(56.67%) | 0(0%) | 0(0%) |
| Resistance is a condition where the treatment of bacterial infections is not successful/ineffective | Correct Answer | 17(42.5%) | 1(2.5%) | 4(80%) | 10(33.33%) | 2(66.67%) | 2(100%) |
|  | Incorrect Answer | 16(40%) | 6(15%) | 1(20%) | 20(66.67%) | 1(33.33%) | 0(0%) |
| The use of antibiotics in low doses in feed for a long time can accelerate the occurrence of antibiotic resistance | Correct Answer | 25(62.5%) | 7(17.5%) | 3(60%) | 26(86.67%) | 2(66.67%) | 1(50%) |
|  | Incorrect Answer | 8(20%) | 0(0%) | 2(40%) | 4(13.33%) | 1(33.33%) | 1(50%) |
| Antibiotic resistance causes ineffective treatment of diseases in livestock, which is detrimental economically | Correct Answer | 27(67.5%) | 6(15%) | 5(100%) | 23(76.67%) | 3(100%) | 2(100%) |
|  | Incorrect Answer | 6(15%) | 1(2.5%) | 0(0%) | 7(23.33%) | 0(0%) | 0(0%) |
| Bacteria that are resistant to antibiotics can contaminate the environment and can render treatment of disease ineffective (including treatment in humans). | Correct Answer | 6(15%) | 1(2.5%) | 2(40%) | 2(6.67%) | 1(33.33%) | 2(100%) |
|  | Incorrect Answer | 27(67.5%) | 6(15%) | 3(60%) | 28(93.33%) | 2(66.67%) | 0(0%) |
| In Indonesia are there laws and regulations to control or control the use of antibiotics? | Correct Answer | 26(65%) | 7(17.5%) | 2(40%) | 28(93.33%) | 2(66.67%) | 1(50%) |
|  | Incorrect Answer | 7(17.5%) | 0(0%) | 3(60%) | 2(6.67%) | 1(33.33%) | 1(50%) |
| Antibiotics that are found to be sold freely in the market without labels and permit numbers clearly violate applicable regulations. | Correct Answer | 22(55%) | 4(10%) | 5(100%) | 16(53.33%) | 3(100%) | 2(100%) |
|  | Incorrect Answer | 11(27.5%) | 3(7.5%) | 0(0%) | 14(46.67%) | 0(0%) | 0(0%) |
| Distribution/sale of antimicrobials/antibiotics for animals must be through authorized persons/institutions in accordance with the law. | Correct Answer | 21(52.5%) | 1(2.5%) | 4(80%) | 15(50%) | 1(33.33%) | 2(100%) |
|  | Incorrect Answer | 12(30%) | 6(15%) | 1(20%) | 15(50%) | 2(66.67%) | 0(0%) |
| *There is no significant difference between demographic variables and the responses to each question. | | | | | | | |

| **Table 2. Knowledge questions from the pre-ban questionnaire with correct (“Yes”) and incorrect (“No” or “Do not know”) answers, presented by education level, farm status, age, and farming experience.** | | | | | | | | | | |
| --- | --- | --- | --- | --- | --- | --- | --- | --- | --- | --- |
|  |  | **Education Level** | | | | | **Statue of Farm** | | **Age** | **Farming Experience** |
| **Question** | **Answers** | **Bachelor** | **Diploma** | **Graduated from High School** | **Graduated from elementary school** | **Middle school graduate** | **Not Registered** | **Registered** | **Age** | **Farming Experience** |
| Antibiotics are drugs that can be used to treat all kinds of diseases? | Correct Answer | 5(71.43%) | 3(37.5%) | 8(53.33%) | 0(0%) | 2(50%) | 11(39.29%) | 7(58.33%) | 45.3(26-72) | 16.4(1-38) |
|  | Incorrect Answer | 2(28.57%) | 5(62.5%) | 7(46.67%) | 6(100%) | 2(50%) | 17(60.71%) | 5(41.67%) | 46.3(26-66) | 16.1(2-37) |
| Disease treatment should use one type of antibiotic in the long term without having to rotate with other types? | Correct Answer | 5(71.43%) | 5(62.5%) | 10(66.67%) | 2(33.33%) | 3(75%) | 15(53.57%) | 10(83.33%) | 46.2(26-72) | 18.2(1-38) |
|  | Incorrect Answer | 2(28.57%) | 3(37.5%) | 5(33.33%) | 4(66.67%) | 1(25%) | 13(46.43%) | 2(16.67%) | 45.3(26-66) | 13(4.5-34) |
| The use of antibiotics for the treatment of disease should be consulted with competent medical personnel | Correct Answer | 5(71.43%) | 4(50%) | 12(80%) | 3(50%) | 3(75%) | 17(60.71%) | 10(83.33%) | 44.8(26-72) | 15.3(1-38) |
|  | Incorrect Answer | 2(28.57%) | 4(50%) | 3(20%) | 3(50%) | 1(25%) | 11(39.29%) | 2(16.67%) | 48.1(26-66) | 18.2(7-37) |
| Antibiotic compounds may not be traded freely (without a prescription/consultation of medical personnel)? | Correct Answer | 4(57.14%) | 2(25%) | 5(33.33%) | 1(16.67%) | 2(50%) | 7(25%) | 7(58.33%) | 45.6(26-72) | 15(1-38) |
|  | Incorrect Answer | 3(42.86%) | 6(75%) | 10(66.67%) | 5(83.33%) | 2(50%) | 21(75%) | 5(41.67%) | 46(26-66) | 16.9(4.5-37) |
| Antibiotic compounds contained in feed should not be of the same type as antibiotics used for treatment in humans? | Correct Answer | 1(14.29%) | 6(75%) | 12(80%) | 5(83.33%) | 2(50%) | 19(67.86%) | 7(58.33%) | 47.4(26-72) | 17.5(2-38) |
|  | Incorrect Answer | 6(85.71%) | 2(25%) | 3(20%) | 1(16.67%) | 2(50%) | 9(32.14%) | 5(41.67%) | 43(26-71) | 14(1-24) |
| The use of antibiotics for treatment should be determined based on the cause of the disease? | Correct Answer | 7(100%) | 7(87.5%) | 13(86.67%) | 3(50%) | 3(75%) | 21(75%) | 12(100%) | 44.5(26-72) | 15.5(1-38) |
|  | Incorrect Answer | 0(0%) | 1(12.5%) | 2(13.33%) | 3(50%) | 1(25%) | 7(25%) | 0(0%) | 52.4(26-66) | 20(7-37) |

| **Table 2. Continued** |  |  |  |  |  |  |  |  |  |  |
| --- | --- | --- | --- | --- | --- | --- | --- | --- | --- | --- |
| Use of antibiotics less than the prescribed dose for treatment can cause antibiotic resistance? | Correct Answer | 3(42.86%) | 2(25%) | 6(40%) | 1(16.67%) | 1(25%) | 8(28.57%) | 5(41.67%) | 45(26-72) | 17.2(1-38) |
|  | Incorrect Answer | 4(57.14%) | 6(75%) | 9(60%) | 5(83.33%) | 3(75%) | 20(71.43%) | 7(58.33%) | 46.3(26-71) | 15.8(2-37) |
| Can the continuous use of antibiotics lead to antibiotic resistance? | Correct Answer | 4(57.14%) | 7(87.5%) | 10(66.67%) | 2(33.33%) | 3(75%) | 16(57.14%) | 10(83.33%) | 45.5(26-72) | 17.2(1-38) |
|  | Incorrect Answer | 3(42.86%) | 1(12.5%) | 5(33.33%) | 4(66.67%) | 1(25%) | 12(42.86%) | 2(16.67%) | 46.6(26-66) | 14.4(2-37) |
| Antibiotics in their use must follow the provisions of the drug stop time? | Correct Answer | 5(71.43%) | 6(75%) | 11(73.33%) | 3(50%) | 2(50%) | 18(64.29%) | 9(75%) | 44.5(26-72) | 16.2(1-38) |
|  | Incorrect Answer | 2(28.57%) | 2(25%) | 4(26.67%) | 3(50%) | 2(50%) | 10(35.71%) | 3(25%) | 48.8(26-71) | 16.5(7-37) |
| Uncontrolled use of antibiotics can lead to antibiotic resistance? | Correct Answer | 6(85.71%) | 5(62.5%) | 7(46.67%) | 1(16.67%) | 3(75%) | 13(46.43%) | 9(75%) | 44(26-72) | 16.3(1-38) |
|  | Incorrect Answer | 1(14.29%) | 3(37.5%) | 8(53.33%) | 5(83.33%) | 1(25%) | 15(53.57%) | 3(25%) | 48.2(26-66) | 16.2(2-37) |
| Resistance is a condition where the treatment of bacterial infections is not successful/ineffective | Correct Answer | 6(85.71%) | 6(75%) | 5(33.33%) | 0(0%) | 1(25%) | 10(35.71%) | 8(66.67%) | 43.2(26-72) | 16.4(1-38) |
|  | Incorrect Answer | 1(14.29%) | 2(25%) | 10(66.67%) | 6(100%) | 3(75%) | 18(64.29%) | 4(33.33%) | 48.1(26-71) | 16.2(2-37) |
| The use of antibiotics in low doses in feed for a long time can accelerate the occurrence of antibiotic resistance | Correct Answer | 5(71.43%) | 5(62.5%) | 12(80%) | 6(100%) | 4(100%) | 24(85.71%) | 8(66.67%) | 45.8(26-71) | 15.3(1-37) |
|  | Incorrect Answer | 2(28.57%) | 3(37.5%) | 3(20%) | 0(0%) | 0(0%) | 4(14.29%) | 4(33.33%) | 46.2(30-72) | 20(10-38) |
| Antibiotic resistance causes ineffective treatment of diseases in livestock, which is detrimental economically | Correct Answer | 7(100%) | 8(100%) | 11(73.33%) | 4(66.67%) | 3(75%) | 21(75%) | 12(100%) | 45.4(26-72) | 15.5(1-38) |
|  | Incorrect Answer | 0(0%) | 0(0%) | 4(26.67%) | 2(33.33%) | 1(25%) | 7(25%) | 0(0%) | 48.1(26-66) | 20.1(4.5-37) |

| **Table 2. Continued** |  |  |  |  |  |  |  |  |  |  |
| --- | --- | --- | --- | --- | --- | --- | --- | --- | --- | --- |
| Bacteria that are resistant to antibiotics can contaminate the environment and can render treatment of disease ineffective (including treatment in humans). | Correct Answer | 3(42.86%) | 2(25%) | 2(13.33%) | 0(0%) | 0(0%) | 2(7.14%) | 5(41.67%) | 44.1(30-57) | 16.9(9-24) |
|  | Incorrect Answer | 4(57.14%) | 6(75%) | 13(86.67%) | 6(100%) | 4(100%) | 26(92.86%) | 7(58.33%) | 46.2(26-72) | 16.1(1-38) |
| In Indonesia are there laws and regulations to control or control the use of antibiotics? | Correct Answer | 3(42.86%) | 6(75%) | 14(93.33%) | 6(100%) | 4(100%) | 26(92.86%) | 7(58.33%) | 46.4(26-72) | 16.1(2-38) |
|  | Incorrect Answer | 4(57.14%) | 2(25%) | 1(6.67%) | 0(0%) | 0(0%) | 2(7.14%) | 5(41.67%) | 43.6(26-57) | 16.9(1-24) |
| Antibiotics that are found to be sold freely in the market without labels and permit numbers clearly violate applicable regulations. | Correct Answer | 6(85.71%) | 5(62.5%) | 10(66.67%) | 3(50%) | 2(50%) | 14(50%) | 12(100%) | 41.9(26-72) | 14.3(1-38) |
|  | Incorrect Answer | 1(14.29%) | 3(37.5%) | 5(33.33%) | 3(50%) | 2(50%) | 14(50%) | 0(0%) | 53.2(34-71) | 19.9(7-37) |
| Distribution/sale of antimicrobials/antibiotics for animals must be through authorized persons/institutions in accordance with the law. | Correct Answer | 6(85.71%) | 5(62.5%) | 7(46.67%) | 3(50%) | 1(25%) | 13(46.43%) | 9(75%) | 45(26-72) | 15.7(1-38) |
|  | Incorrect Answer | 1(14.29%) | 3(37.5%) | 8(53.33%) | 3(50%) | 3(75%) | 15(53.57%) | 3(25%) | 46.9(26-71) | 17(4.5-37) |
| *There is no significant difference between demographic variables and the responses to each question. | | | | | | | | | | |

| **Table 3. Attitude questions from the pre-ban questionnaire, with correct (“Yes”) and incorrect (“No” or “Do not know”) responses, presented by gender and farm position.** | | | | | | | |
| --- | --- | --- | --- | --- | --- | --- | --- |
|  |  | **Gender** | | **Position in Farm** | | | |
| **Question** | **Answers** | **Male** | **Female** | **Manager** | **Owner** | **head of the cage** | **health expert** |
| Do you agree that antibiotics can be used for all types of infectious diseases: | Correct Answer | 10(25%) | 3(7.5%) | 4(80%) | 7(23.33%) | 1(33.33%) | 1(50%) |
|  | Incorrect Answer | 23(57.5%) | 4(10%) | 1(20%) | 23(76.67%) | 2(66.67%) | 1(50%) |
| Do you agree that using one type of antibiotic should not be used for a long time? | Correct Answer | 19(47.5%) | 4(10%) | 4(80%) | 14(46.67%) | 3(100%) | 2(100%) |
|  | Incorrect Answer | 14(35%) | 3(7.5%) | 1(20%) | 16(53.33%) | 0(0%) | 0(0%) |
| Do you agree that the use of antibiotics for treatment does not have to be consulted with medical personnel | Correct Answer | 15(37.5%) | 4(10%) | 1(20%) | 15(50%) | 2(66.67%) | 1(50%) |
|  | Incorrect Answer | 18(45%) | 3(7.5%) | 4(80%) | 15(50%) | 1(33.33%) | 1(50%) |
| Do you agree that antibiotics can be traded freely: | Correct Answer | 10(25%) | 1(2.5%) | 3(60%) | 6(20%) | 2(66.67%) | 0(0%) |
|  | Incorrect Answer | 23(57.5%) | 6(15%) | 2(40%) | 24(80%) | 1(33.33%) | 2(100%) |
| Do you agree that the use of antibiotics in feed should not contain the types of antibiotics used for treatment in humans | Correct Answer | 13(32.5%) | 3(7.5%) | 2(40%) | 10(33.33%) | 2(66.67%) | 2(100%) |
|  | Incorrect Answer | 20(50%) | 4(10%) | 3(60%) | 20(66.67%) | 1(33.33%) | 0(0%) |
| Do you agree that the use of antibiotics for the treatment of disease must be based on the cause | Correct Answer | 29(72.5%) | 5(12.5%) | 5(100%) | 24(80%) | 3(100%) | 2(100%) |
|  | Incorrect Answer | 4(10%) | 2(5%) | 0(0%) | 6(20%) | 0(0%) | 0(0%) |
| Do you agree that using antibiotics less than the prescribed dose can cause antibiotic resistance | Correct Answer | 17(42.5%) | 2(5%) | 3(60%) | 12(40%) | 2(66.67%) | 2(100%) |
|  | Incorrect Answer | 16(40%) | 5(12.5%) | 2(40%) | 18(60%) | 1(33.33%) | 0(0%) |
| Do you agree that the continuous use of antibiotics can lead to resistance? | Correct Answer | 22(55%) | 5(12.5%) | 5(100%) | 17(56.67%) | 3(100%) | 2(100%) |
|  | Incorrect Answer | 11(27.5%) | 2(5%) | 0(0%) | 13(43.33%) | 0(0%) | 0(0%) |
| Do you agree that the use of antibiotics does not have to comply with the provisions for stopping the drug? | Correct Answer | 24(60%) | 4(10%) | 5(100%) | 20(66.67%) | 2(66.67%) | 1(50%) |
|  | Incorrect Answer | 9(22.5%) | 3(7.5%) | 0(0%) | 10(33.33%) | 1(33.33%) | 1(50%) |

| **Table 3. Continued** | | | | | | | |
| --- | --- | --- | --- | --- | --- | --- | --- |
| Do you agree that uncontrolled use of antibiotics can lead to antibiotic resistance? | Correct Answer | 22(55%) | 4(10%) | 5(100%) | 16(53.33%) | 3(100%) | 2(100%) |
|  | Incorrect Answer | 11(27.5%) | 3(7.5%) | 0(0%) | 14(46.67%) | 0(0%) | 0(0%) |
| Do you agree that resistance causes failure to treat bacterial infections? | Correct Answer | 20(50%) | 1(2.5%) | 4(80%) | 13(43.33%) | 2(66.67%) | 2(100%) |
|  | Incorrect Answer | 13(32.5%) | 6(15%) | 1(20%) | 17(56.67%) | 1(33.33%) | 0(0%) |
| Do you agree that antibiotics used as feed additives (with doses used below the treatment dose) can accelerate the occurrence of resistance | Correct Answer | 23(57.5%) | 7(17.5%) | 3(60%) | 24(80%) | 2(66.67%) | 1(50%) |
|  | Incorrect Answer | 10(25%) | 0(0%) | 2(40%) | 6(20%) | 1(33.33%) | 1(50%) |
| Do you agree that the treatment of disease with ineffective antibiotics causes economic losses? | Correct Answer | 31(77.5%) | 7(17.5%) | 5(100%) | 28(93.33%) | 3(100%) | 2(100%) |
|  | Incorrect Answer | 2(5%) | 0(0%) | 0(0%) | 2(6.67%) | 0(0%) | 0(0%) |
| Do you agree that bacteria that are resistant to antibiotics can contaminate the environment? | Correct Answer | 7(17.5%) | 1(2.5%) | 2(40%) | 3(10%) | 1(33.33%) | 2(100%) |
|  | Incorrect Answer | 26(65%) | 6(15%) | 3(60%) | 27(90%) | 2(66.67%) | 0(0%) |
| The use of antibiotics needs to be regulated in regulations or laws | Correct Answer | 22(55%) | 4(10%) | 4(80%) | 18(60%) | 3(100%) | 1(50%) |
|  | Incorrect Answer | 11(27.5%) | 3(7.5%) | 1(20%) | 12(40%) | 0(0%) | 1(50%) |
| Every over-the-counter antibiotic needs to get a permit and a clear label | Correct Answer | 26(65%) | 6(15%) | 5(100%) | 22(73.33%) | 3(100%) | 2(100%) |
|  | Incorrect Answer | 7(17.5%) | 1(2.5%) | 0(0%) | 8(26.67%) | 0(0%) | 0(0%) |
| Do you agree that the distribution/sale of antimicrobials/antibiotics for animals must go through a permit from the department of agriculture. | Correct Answer | 29(72.5%) | 5(12.5%) | 4(80%) | 25(83.33%) | 3(100%) | 2(100%) |
|  | Incorrect Answer | 4(10%) | 2(5%) | 1(20%) | 5(16.67%) | 0(0%) | 0(0%) |
| *There is no significant difference between demographic variables and the responses to each question. | | | | | | | |

| **Table 4. Attitude questions from the pre-ban questionnaire with correct (“Yes”) and incorrect (“No” or “Do not know”) answers, presented by education level, farm status, age, and farming experience.** | | | | | | | | | | |
| --- | --- | --- | --- | --- | --- | --- | --- | --- | --- | --- |
|  |  | **Education Level** | | | | | **Statue of Farm** | | **Age** | **Farming Experience** |
| **Question** | **Answers** | **Bachelor** | **Diploma** | **Graduated from High School** | **Graduated from elementary school** | **Middle school graduate** | **Not Registered** | **Registered** | **Age** | **Farming Experience** |
| Do you agree that antibiotics can be used for all types of infectious diseases: | Correct Answer | 3(42.86%) | 2(25%) | 6(40%) | 0(0%) | 2(50%) | 7(25%) | 6(50%) | 43.9(26-71) | 15.2(1-24) |
|  | Incorrect Answer | 4(57.14%) | 6(75%) | 9(60%) | 6(100%) | 2(50%) | 21(75%) | 6(50%) | 46.8(26-72) | 16.8(2-38) |
| Do you agree that using one type of antibiotic should not be used for a long time? | Correct Answer | 5(71.43%) | 6(75%) | 10(66.67%) | 2(33.33%) | 0(0%) | 13(46.43%) | 10(83.33%) | 42.2(26-72) | 15.7(1-38) |
|  | Incorrect Answer | 2(28.57%) | 2(25%) | 5(33.33%) | 4(66.67%) | 4(100%) | 15(53.57%) | 2(16.67%) | 50.8(26-71) | 17.1(4.5-37) |
| Do you agree that the use of antibiotics for treatment does not have to be consulted with medical personnel | Correct Answer | 3(42.86%) | 3(37.5%) | 8(53.33%) | 3(50%) | 2(50%) | 14(50%) | 5(41.67%) | 46.4(26-64) | 16.6(2-37) |
|  | Incorrect Answer | 4(57.14%) | 5(62.5%) | 7(46.67%) | 3(50%) | 2(50%) | 14(50%) | 7(58.33%) | 45.4(26-72) | 16(1-38) |
| Do you agree that antibiotics can be traded freely: | Correct Answer | 2(28.57%) | 3(37.5%) | 3(20%) | 1(16.67%) | 2(50%) | 5(17.86%) | 6(50%) | 45.5(26-71) | 15.4(1-36) |
|  | Incorrect Answer | 5(71.43%) | 5(62.5%) | 12(80%) | 5(83.33%) | 2(50%) | 23(82.14%) | 6(50%) | 46(26-72) | 16.6(4.5-38) |
| Do you agree that the use of antibiotics in feed should not contain the types of antibiotics used for treatment in humans | Correct Answer | 4(57.14%) | 2(25%) | 6(40%) | 3(50%) | 1(25%) | 10(35.71%) | 6(50%) | 45.4(26-66) | 15.3(1-36) |
|  | Incorrect Answer | 3(42.86%) | 6(75%) | 9(60%) | 3(50%) | 3(75%) | 18(64.29%) | 6(50%) | 46.2(26-72) | 16.9(2-38) |

| **Table 4. Continued** | | | | | | | | | | |
| --- | --- | --- | --- | --- | --- | --- | --- | --- | --- | --- |
| Do you agree that the use of antibiotics for the treatment of disease must be based on the cause | Correct Answer | 7(100%) | 8(100%) | 13(86.67%) | 3(50%) | 3(75%) | 22(78.57%) | 12(100%) | 44.5(26-72) | 15.4(1-38) |
|  | Incorrect Answer | 0(0%) | 0(0%) | 2(13.33%) | 3(50%) | 1(25%) | 6(21.43%) | 0(0%) | 53.7(26-66) | 21(7-37) |
| Do you agree that using antibiotics less than the prescribed dose can cause antibiotic resistance | Correct Answer | 5(71.43%) | 4(50%) | 7(46.67%) | 2(33.33%) | 1(25%) | 11(39.29%) | 8(66.67%) | 43.8(26-72) | 15.4(1-38) |
|  | Incorrect Answer | 2(28.57%) | 4(50%) | 8(53.33%) | 4(66.67%) | 3(75%) | 17(60.71%) | 4(33.33%) | 47.8(26-71) | 17(4.5-37) |
| Do you agree that the continuous use of antibiotics can lead to resistance? | Correct Answer | 5(71.43%) | 7(87.5%) | 9(60%) | 3(50%) | 3(75%) | 16(57.14%) | 11(91.67%) | 45.1(26-72) | 16.1(1-38) |
|  | Incorrect Answer | 2(28.57%) | 1(12.5%) | 6(40%) | 3(50%) | 1(25%) | 12(42.86%) | 1(8.33%) | 47.5(26-66) | 16.5(4.5-37) |
| Do you agree that the use of antibiotics does not have to comply with the provisions for stopping the drug? | Correct Answer | 4(57.14%) | 7(87.5%) | 12(80%) | 3(50%) | 2(50%) | 18(64.29%) | 10(83.33%) | 44(26-72) | 14.9(1-38) |
|  | Incorrect Answer | 3(42.86%) | 1(12.5%) | 3(20%) | 3(50%) | 2(50%) | 10(35.71%) | 2(16.67%) | 50.2(32-65) | 19.3(9-37) |
| Do you agree that uncontrolled use of antibiotics can lead to antibiotic resistance? | Correct Answer | 6(85.71%) | 6(75%) | 9(60%) | 2(33.33%) | 3(75%) | 15(53.57%) | 11(91.67%) | 44(26-72) | 15.6(1-38) |
|  | Incorrect Answer | 1(14.29%) | 2(25%) | 6(40%) | 4(66.67%) | 1(25%) | 13(46.43%) | 1(8.33%) | 49.4(26-66) | 17.4(4.5-37) |
| Do you agree that resistance causes failure to treat bacterial infections? | Correct Answer | 6(85.71%) | 6(75%) | 6(40%) | 0(0%) | 3(75%) | 13(46.43%) | 8(66.67%) | 45(26-72) | 16.5(1-38) |
|  | Incorrect Answer | 1(14.29%) | 2(25%) | 9(60%) | 6(100%) | 1(25%) | 15(53.57%) | 4(33.33%) | 46.9(26-66) | 15.9(2-37) |
| Do you agree that antibiotics used as feed additives (with doses used below the treatment dose) can accelerate the occurrence of resistance | Correct Answer | 5(71.43%) | 5(62.5%) | 11(73.33%) | 6(100%) | 3(75%) | 22(78.57%) | 8(66.67%) | 46(26-71) | 15.5(1-37) |
|  | Incorrect Answer | 2(28.57%) | 3(37.5%) | 4(26.67%) | 0(0%) | 1(25%) | 6(21.43%) | 4(33.33%) | 45.4(30-72) | 18.4(7.5-38) |

| **Table 4. Continued** | | | | | | | | | | |
| --- | --- | --- | --- | --- | --- | --- | --- | --- | --- | --- |
| Do you agree that the treatment of disease with ineffective antibiotics causes economic losses? | Correct Answer | 7(100%) | 8(100%) | 14(93.33%) | 6(100%) | 3(75%) | 26(92.86%) | 12(100%) | 45.8(26-72) | 16(1-38) |
|  | Incorrect Answer | 0(0%) | 0(0%) | 1(6.67%) | 0(0%) | 1(25%) | 2(7.14%) | 0(0%) | 47.5(32-63) | 20.8(4.5-37) |
| Do you agree that bacteria that are resistant to antibiotics can contaminate the environment? | Correct Answer | 3(42.86%) | 2(25%) | 2(13.33%) | 0(0%) | 1(25%) | 3(10.71%) | 5(41.67%) | 47.5(30-71) | 17.2(9-24) |
|  | Incorrect Answer | 4(57.14%) | 6(75%) | 13(86.67%) | 6(100%) | 3(75%) | 25(89.29%) | 7(58.33%) | 45.5(26-72) | 16(1-38) |
| The use of antibiotics needs to be regulated in regulations or laws | Correct Answer | 4(57.14%) | 8(100%) | 10(66.67%) | 2(33.33%) | 2(50%) | 17(60.71%) | 9(75%) | 43.8(26-72) | 14.7(1-38) |
|  | Incorrect Answer | 3(42.86%) | 0(0%) | 5(33.33%) | 4(66.67%) | 2(50%) | 11(39.29%) | 3(25%) | 49.7(26-71) | 19.1(7-37) |
| Every over-the-counter antibiotic needs to get a permit and a clear label | Correct Answer | 6(85.71%) | 6(75%) | 13(86.67%) | 3(50%) | 4(100%) | 20(71.43%) | 12(100%) | 43.7(26-72) | 15.9(1-38) |
|  | Incorrect Answer | 1(14.29%) | 2(25%) | 2(13.33%) | 3(50%) | 0(0%) | 8(28.57%) | 0(0%) | 54.6(32-66) | 17.7(4.5-36) |
| Do you agree that the distribution/sale of antimicrobials/antibiotics for animals must go through a permit from the department of agriculture. | Correct Answer | 7(100%) | 8(100%) | 12(80%) | 3(50%) | 4(100%) | 23(82.14%) | 11(91.67%) | 45.4(26-72) | 16.6(1-38) |
|  | Incorrect Answer | 0(0%) | 0(0%) | 3(20%) | 3(50%) | 0(0%) | 5(17.86%) | 1(8.33%) | 48.3(32-66) | 14.2(4.5-34) |
| *There is no significant difference between demographic variables and the responses to each question. | | | | | | | | | | |

| **Table 5. Practice questions from the pre-ban questionnaire, with correct (“Yes”) and incorrect (“No” or “Do not know”) responses, presented by gender and farm position.** | | | | | | | |
| --- | --- | --- | --- | --- | --- | --- | --- |
|  |  | **Gender** | | **Position in Farm** | | | |
| **Question** | **Answers** | **Male** | **Female** | **Manager** | **Owner** | **head of the cage** | **health expert** |
| Do you give antibiotic compounds or drugs to every sick animal? | Correct Answer | 12(30%) | 3(7.5%) | 3(60%) | 9(30%) | 2(66.67%) | 1(50%) |
|  | Incorrect Answer | 21(52.5%) | 4(10%) | 2(40%) | 21(70%) | 1(33.33%) | 1(50%) |
| Do you rotate the use of antibiotic compounds? | Correct Answer | 19(47.5%) | 5(12.5%) | 4(80%) | 15(50%) | 3(100%) | 2(100%) |
|  | Incorrect Answer | 14(35%) | 2(5%) | 1(20%) | 15(50%) | 0(0%) | 0(0%) |
| Is it always consulted with medical personnel when using antibiotics for the treatment of disease? | Correct Answer | 6(15%) | 3(7.5%) | 2(40%) | 5(16.67%) | 2(66.67%) | 0(0%) |
|  | Incorrect Answer | 27(67.5%) | 4(10%) | 3(60%) | 25(83.33%) | 1(33.33%) | 2(100%) |
| Can you always buy antibiotics easily (without a prescription/consulting a medical professional)? | Correct Answer | 3(7.5%) | 1(2.5%) | 1(20%) | 1(3.33%) | 2(66.67%) | 0(0%) |
|  | Incorrect Answer | 30(75%) | 6(15%) | 4(80%) | 29(96.67%) | 1(33.33%) | 2(100%) |
| Did you consider the types of antibiotics contained in the feed? | Correct Answer | 16(40%) | 3(7.5%) | 3(60%) | 13(43.33%) | 1(33.33%) | 2(100%) |
|  | Incorrect Answer | 17(42.5%) | 4(10%) | 2(40%) | 17(56.67%) | 2(66.67%) | 0(0%) |
| Do you use antibiotics only for diseases caused by bacteria? | Correct Answer | 10(25%) | 3(7.5%) | 5(100%) | 3(10%) | 3(100%) | 2(100%) |
|  | Incorrect Answer | 23(57.5%) | 4(10%) | 0(0%) | 27(90%) | 0(0%) | 0(0%) |
| Do you consider the dosage in using antibiotics for the treatment of disease? | Correct Answer | 25(62.5%) | 5(12.5%) | 5(100%) | 20(66.67%) | 3(100%) | 2(100%) |
|  | Incorrect Answer | 8(20%) | 2(5%) | 0(0%) | 10(33.33%) | 0(0%) | 0(0%) |
| Do you use antibiotics according to the prescribed dosage for the treatment of disease? | Correct Answer | 23(57.5%) | 4(10%) | 5(100%) | 17(56.67%) | 3(100%) | 2(100%) |
|  | Incorrect Answer | 10(25%) | 3(7.5%) | 0(0%) | 13(43.33%) | 0(0%) | 0(0%) |
| Do you use antibiotics for treatment according to the specified time? | Correct Answer | 19(47.5%) | 3(7.5%) | 5(100%) | 13(43.33%) | 2(66.67%) | 2(100%) |
|  | Incorrect Answer | 14(35%) | 4(10%) | 0(0%) | 17(56.67%) | 1(33.33%) | 0(0%) |

| **Table 5. Continued** | | | | | | | |
| --- | --- | --- | --- | --- | --- | --- | --- |
| Do you use compounds or antibiotic drugs according to the rules for use? | Correct Answer | 19(47.5%) | 4(10%) | 5(100%) | 13(43.33%) | 3(100%) | 2(100%) |
|  | Incorrect Answer | 14(35%) | 3(7.5%) | 0(0%) | 17(56.67%) | 0(0%) | 0(0%) |
| Has your farm ever experienced a condition where the treatment of bacterial infection using antibiotics was ineffective/unsuccessful? | Correct Answer | 5(12.5%) | 1(2.5%) | 1(20%) | 2(6.67%) | 1(33.33%) | 2(100%) |
|  | Incorrect Answer | 28(70%) | 6(15%) | 4(80%) | 28(93.33%) | 2(66.67%) | 0(0%) |
| Do you use commercial feed containing antibiotics? | Correct Answer | 30(75%) | 6(15%) | 3(60%) | 30(100%) | 2(66.67%) | 1(50%) |
|  | Incorrect Answer | 3(7.5%) | 1(2.5%) | 2(40%) | 0(0%) | 1(33.33%) | 1(50%) |
| Do you have experience with using antibiotics that are not effective in treating diseases, and this is a loss because there are additional costs | Correct Answer | 21(52.5%) | 7(17.5%) | 3(60%) | 23(76.67%) | 2(66.67%) | 0(0%) |
|  | Incorrect Answer | 12(30%) | 0(0%) | 2(40%) | 7(23.33%) | 1(33.33%) | 2(100%) |
| Do you pay attention to the regulations that apply in buying and using antibiotics | Correct Answer | 5(12.5%) | 3(7.5%) | 0(0%) | 4(13.33%) | 2(66.67%) | 2(100%) |
|  | Incorrect Answer | 28(70%) | 4(10%) | 5(100%) | 26(86.67%) | 1(33.33%) | 0(0%) |
| Have you ever used unlabeled/unlicensed antibiotics? | Correct Answer | 29(72.5%) | 5(12.5%) | 4(80%) | 25(83.33%) | 3(100%) | 2(100%) |
|  | Incorrect Answer | 4(10%) | 2(5%) | 1(20%) | 5(16.67%) | 0(0%) | 0(0%) |
| Have you ever bought antibiotics at a company/drugstore/another place that doesn't have an official permit? | Correct Answer | 24(60%) | 4(10%) | 4(80%) | 19(63.33%) | 3(100%) | 2(100%) |
|  | Incorrect Answer | 9(22.5%) | 3(7.5%) | 1(20%) | 11(36.67%) | 0(0%) | 0(0%) |
| *There is no significant difference between demographic variables and the responses to each question. | | | | | | | |

| **Table 6. Practice questions from the pre-ban questionnaire with correct (“Yes”) and incorrect (“No” or “Do not know”) answers, presented by education level, farm status, age, and farming experience.** | | | | | | | | | | |
| --- | --- | --- | --- | --- | --- | --- | --- | --- | --- | --- |
|  |  | **Education Level** | | | | | **Statue of Farm** | | **Age** | **Farming Experience** |
| **Question** | **Answers** | **Bachelor** | **Diploma** | **Graduated from High School** | **Graduated from elementary school** | **Middle school graduate** | **Not Registered** | **Registered** | **Age** | **Farming Experience** |
| Do you give antibiotic compounds or drugs to every sick animal? | Correct Answer | 4(57.14%) | 3(37.5%) | 7(46.67%) | 0(0%) | 1(25%) | 8(28.57%) | 7(58.33%) | 43.3(26-72) | 15.9(1-38) |
|  | Incorrect Answer | 3(42.86%) | 5(62.5%) | 8(53.33%) | 6(100%) | 3(75%) | 20(71.43%) | 5(41.67%) | 47.4(30-66) | 16.5(2-37) |
| Do you rotate the use of antibiotic compounds? | Correct Answer | 5(71.43%) | 5(62.5%) | 11(73.33%) | 2(33.33%) | 1(25%) | 13(46.43%) | 11(91.67%) | 42.5(26-72) | 15.6(1-38) |
|  | Incorrect Answer | 2(28.57%) | 3(37.5%) | 4(26.67%) | 4(66.67%) | 3(75%) | 15(53.57%) | 1(8.33%) | 50.9(30-66) | 17.2(4.5-37) |
| Is it always consulted with medical personnel when using antibiotics for the treatment of disease? | Correct Answer | 0(0%) | 3(37.5%) | 5(33.33%) | 1(16.67%) | 0(0%) | 4(14.29%) | 5(41.67%) | 42.3(30-51) | 13.8(2-28) |
|  | Incorrect Answer | 7(100%) | 5(62.5%) | 10(66.67%) | 5(83.33%) | 4(100%) | 24(85.71%) | 7(58.33%) | 46.9(26-72) | 17(1-38) |
| Can you always buy antibiotics easily (without a prescription/consulting a medical professional)? | Correct Answer | 0(0%) | 1(12.5%) | 3(20%) | 0(0%) | 0(0%) | 1(3.57%) | 3(25%) | 42.5(30-63) | 17.8(10-36) |
|  | Incorrect Answer | 7(100%) | 7(87.5%) | 12(80%) | 6(100%) | 4(100%) | 27(96.43%) | 9(75%) | 46.2(26-72) | 16.1(1-38) |
| Did you consider the types of antibiotics contained in the feed? | Correct Answer | 4(57.14%) | 3(37.5%) | 8(53.33%) | 2(33.33%) | 2(50%) | 11(39.29%) | 8(66.67%) | 42.5(29-71) | 14.2(2-28) |
|  | Incorrect Answer | 3(42.86%) | 5(62.5%) | 7(46.67%) | 4(66.67%) | 2(50%) | 17(60.71%) | 4(33.33%) | 48.9(26-72) | 18.1(1-38) |

| **Table 6. Continued** | | | | | | | | | | |
| --- | --- | --- | --- | --- | --- | --- | --- | --- | --- | --- |
| Do you use antibiotics only for diseases caused by bacteria? | Correct Answer | 5(71.43%) | 5(62.5%) | 3(20%) | 0(0%) | 0(0%) | 3(10.71%) | 10(83.33%) | 40.6(26-57) | 15.4(1-24) |
|  | Incorrect Answer | 2(28.57%) | 3(37.5%) | 12(80%) | 6(100%) | 4(100%) | 25(89.29%) | 2(16.67%) | 48.4(26-72) | 16.7(2-38) |
| Do you consider the dosage in using antibiotics for the treatment of disease? | Correct Answer | 7(100%) | 7(87.5%) | 11(73.33%) | 3(50%) | 2(50%) | 18(64.29%) | 12(100%) | 45.1(26-72) | 16.7(1-38) |
|  | Incorrect Answer | 0(0%) | 1(12.5%) | 4(26.67%) | 3(50%) | 2(50%) | 10(35.71%) | 0(0%) | 48.1(26-71) | 15.1(7-28) |
| Do you use antibiotics according to the prescribed dosage for the treatment of disease? | Correct Answer | 6(85.71%) | 7(87.5%) | 10(66.67%) | 2(33.33%) | 2(50%) | 15(53.57%) | 12(100%) | 45(26-72) | 16.1(1-38) |
|  | Incorrect Answer | 1(14.29%) | 1(12.5%) | 5(33.33%) | 4(66.67%) | 2(50%) | 13(46.43%) | 0(0%) | 47.8(26-71) | 16.5(7-34) |
| Do you use antibiotics for treatment according to the specified time? | Correct Answer | 5(71.43%) | 6(75%) | 8(53.33%) | 3(50%) | 0(0%) | 11(39.29%) | 11(91.67%) | 43.2(26-72) | 16.1(1-38) |
|  | Incorrect Answer | 2(28.57%) | 2(25%) | 7(46.67%) | 3(50%) | 4(100%) | 17(60.71%) | 1(8.33%) | 49.2(26-71) | 16.5(7-37) |
| Do you use compounds or antibiotic drugs according to the rules for use? | Correct Answer | 6(85.71%) | 6(75%) | 8(53.33%) | 1(16.67%) | 2(50%) | 11(39.29%) | 12(100%) | 42.9(26-63) | 15.6(1-37) |
|  | Incorrect Answer | 1(14.29%) | 2(25%) | 7(46.67%) | 5(83.33%) | 2(50%) | 17(60.71%) | 0(0%) | 49.9(26-72) | 17.1(7-38) |
| Has your farm ever experienced a condition where the treatment of bacterial infection using antibiotics was ineffective/unsuccessful? | Correct Answer | 2(28.57%) | 0(0%) | 3(20%) | 0(0%) | 1(25%) | 2(7.14%) | 4(33.33%) | 44.2(32-63) | 17.4(4.5-37) |
|  | Incorrect Answer | 5(71.43%) | 8(100%) | 12(80%) | 6(100%) | 3(75%) | 26(92.86%) | 8(66.67%) | 46.2(26-72) | 16.1(1-38) |

| **Table 6. Continued** | | | | | | | | | | |
| --- | --- | --- | --- | --- | --- | --- | --- | --- | --- | --- |
| Do you use commercial feed containing antibiotics? | Correct Answer | 5(71.43%) | 7(87.5%) | 14(93.33%) | 6(100%) | 4(100%) | 28(100%) | 8(66.67%) | 47.1(26-72) | 17(2-38) |
|  | Incorrect Answer | 2(28.57%) | 1(12.5%) | 1(6.67%) | 0(0%) | 0(0%) | 0(0%) | 4(33.33%) | 34.8(26-48) | 10(1-20) |
| Do you have experience with using antibiotics that are not effective in treating diseases, and this is a loss because there are additional costs | Correct Answer | 3(42.86%) | 7(87.5%) | 12(80%) | 4(66.67%) | 2(50%) | 21(75%) | 7(58.33%) | 45.9(26-72) | 16.9(2-38) |
|  | Incorrect Answer | 4(57.14%) | 1(12.5%) | 3(20%) | 2(33.33%) | 2(50%) | 7(25%) | 5(41.67%) | 45.8(26-66) | 14.7(1-37) |
| Do you pay attention to the regulations that apply in buying and using antibiotics | Correct Answer | 3(42.86%) | 2(25%) | 3(20%) | 0(0%) | 0(0%) | 4(14.29%) | 4(33.33%) | 41.6(26-57) | 16.1(9-24) |
|  | Incorrect Answer | 4(57.14%) | 6(75%) | 12(80%) | 6(100%) | 4(100%) | 24(85.71%) | 8(66.67%) | 46.9(26-72) | 16.3(1-38) |
| Have you ever used unlabeled/unlicensed antibiotics? | Correct Answer | 7(100%) | 5(62.5%) | 14(93.33%) | 5(83.33%) | 3(75%) | 23(82.14%) | 11(91.67%) | 45.2(26-72) | 15.9(1-38) |
|  | Incorrect Answer | 0(0%) | 3(37.5%) | 1(6.67%) | 1(16.67%) | 1(25%) | 5(17.86%) | 1(8.33%) | 49.5(36-71) | 18.3(10-28) |
| Have you ever bought antibiotics at a company/drugstore/another place that doesn't have an official permit? | Correct Answer | 7(100%) | 6(75%) | 10(66.67%) | 3(50%) | 2(50%) | 17(60.71%) | 11(91.67%) | 45.1(26-72) | 15.6(1-38) |
|  | Incorrect Answer | 0(0%) | 2(25%) | 5(33.33%) | 3(50%) | 2(50%) | 11(39.29%) | 1(8.33%) | 47.6(26-71) | 17.9(4.5-37) |
| *There is no significant difference between demographic variables and the responses to each question. | | | | | | | | | | |

| **Table 7. Questions from the post-ban questionnaire with responses, categorized by gender and farm position.** | | | |
| --- | --- | --- | --- |
| **Question** | **Response(s)** | **Gender** | |
|  |  | **Male** | **Female** |
| **Do you currently use antibiotics on your farm?** | Yes (%) | 43 (86.0%) | 4 (80.0%) |
|  | No (%) | 5 (10.0%) | 1 (20.0%) |
|  | Don’t know (%) | 2 (4.0%) | 0 (0.0%) |
| **Are you aware of the colistin ban in animals in Indonesia?** | Yes (%) | 9 (18.0%) | 1 (20.0%) |
|  | No (%) | 18 (36.0%) | 2 (40.0%) |
|  | Don't Know (%) | 23 (46.0%) | 2 (40.0%) |
| **Do you use the antibiotic colistin in your layer farm?** | Yes (%) | 0 (0%) | 0 (0%) |
|  | No (%) | 33 (66.0%) | 2 (40.0%) |
|  | Don't Know (%) | 17 (34.0%) | 3 (60.0%) |
| **Who is responsible for deciding or permitting the use of drugs, especially antibiotics on your farm?** | Veterinarian | 3 (75%) | 1 (25%) |
|  | Owner | 32 (91.4%) | 3 (8.6%) |
|  | Farmworker | 2 (100%) | 0 (0%) |
|  | Other | 17 (85%) | 3 (15%) |
| **Based on whose consideration is the use of antibiotics in livestock carried out?** | Veterinarian | 7 (77.8%) | 2 (22.2%) |
|  | Drugfeed Technical Service | 19 (86.4%) | 3 (13.6%) |
|  | Lesson Learnt (owner) | 12 (80%) | 3 (20%) |
|  | Other | 29 (85.3%) | 5 (14.7%) |
| **Where do the drugs, especially antibiotics used on your farm, come from?** | Farmworker | 0 (0%) | 1 (100%) |
|  | Owner | 3 (60%) | 40 (76.9%) |
|  | Other | 1 (20%) | 7 (80%) |
|  | With Veterinary Consultation | 1 (20%) | 4 (80%) |
| *There is no significant difference between demographic variables and the responses to each question. | | | |

| **Table 8. Questions from the post-ban questionnaire with responses, presented by education level.** | | | | | | | |
| --- | --- | --- | --- | --- | --- | --- | --- |
| **Question** | **Response(s)** | **Education level** | | | | | |
|  |  | **No School** | **Elementary** | **Junior High** | **Senior High** | **Diploma/Bachelor** | **Master** |
| **Do you currently use antibiotics on your farm?** | Yes (%) | 1 (50.0%) | 4 (66.7%) | 9 (81.8%) | 24 (92.3%) | 7 (87.5%) | 2 (100%) |
|  | No (%) | 1 (50.0%) | 1 (16.7%) | 2 (18.2%) | 2 (7.7%) | 0 (0%) | 0 (0%) |
|  | Don't Know (%) | 0 (0%) | 1 (16.7%) | 0 (0%) | 0 (0%) | 1 (12.5%) | 0 (0%) |
| **Are you aware of the colistin ban in animals in Indonesia?** | Yes (%) | 1 (50.0%) | 0 (0%) | 1 (9.1%) | 6 (23.1%) | 2 (25.0%) | 0 (0%) |
|  | No (%) | 1 (50.0%) | 2 (33.3%) | 3 (27.3%) | 12 (46.2%) | 2 (25.0%) | 0 (0%) |
|  | Don't Know (%) | 0 (0%) | 4 (66.7%) | 7 (63.6%) | 8 (30.8%) | 4 (50.0%) | 2 (100%) |
| **Do you use the antibiotic colistin in your layer farm?** | Yes (%) | 0 (0%) | 0 (0%) | 0 (0%) | 0 (0%) | 0 (0%) | 0 (0%) |
|  | No (%) | 2 (100%) | 4 (66.7%) | 7 (63.6%) | 17 (65.4%) | 5 (62.5%) | 0 (0%) |
|  | Don't Know (%) | 0 (0%) | 2 (33.3%) | 4 (36.4%) | 9 (34.6%) | 3 (37.5%) | 2 (100%) |
| **Who is responsible for deciding or permitting the use of drugs, especially antibiotics on your farm?** | Veterinarian | 0 (0%) | 0 (0%) | 0 (0%) | 2 (50%) | 0 (0%) | 0 (0%) |
|  | Owner | 0 (0%) | 3 (8.6%) | 5 (14.3%) | 15 (42.9%) | 6 (17.1%) | 2 (5.7%) |
|  | Farmworker | 0 (0%) | 1 (50%) | 0 (0%) | 1 (50%) | 0 (0%) | 0 (0%) |
|  | Other | 1 (5%) | 1 (5%) | 5 (25%) | 11 (55%) | 2 (10%) | 1 (5%) |
| **Based on whose consideration is the use of antibiotics in livestock carried out?** | Veterinarian | 0 (0%) | 0 (0%) | 0 (0%) | 5 (55.6%) | 2 (22.2%) | 2 (22.2%) |
|  | Drug-feed Technical Service | 2 (9.1%) | 4 (18.2%) | 7 (31.8%) | 10 (45.5%) | 5 (22.7%) | 2 (9.1%) |
|  | Lesson Learnt (owner) | 0 (0%) | 2 (13.3%) | 4 (26.7%) | 8 (53.3%) | 4 (26.7%) | 0 (0%) |
|  | Other | 2 (5.9%) | 5 (14.7%) | 8 (23.5%) | 23 (67.6%) | 6 (17.6%) | 0 (0%) |
| **Where do the drugs, especially antibiotics used on your farm, come from?** | Farmworker | 0 (0%) | 0 (0%) | 0 (0%) | 1 (100%) | 0 (0%) | 0 (0%) |
|  | Owner | 2 (3.8%) | 6 (11.5%) | 11 (21.2%) | 19 (36.5%) | 7 (13.5%) | 2 (3.8%) |
|  | Other | 0 (0%) | 2 (20%) | 2 (20%) | 4 (40%) | 1 (10%) | 0 (0%) |
|  | With Veterinary Consultation | 0 (0%) | 0 (0%) | 0 (0%) | 4 (80%) | 2 (40%) | 0 (0%) |
| *There is no significant difference between demographic variables and the responses to each question. | | | | | | | |

| **Table 9. Questions from the post-ban questionnaire with responses, presented by education level, mean age, mean farming experience, farm role, and farm registration status.** | | | | | | | | |
| --- | --- | --- | --- | --- | --- | --- | --- | --- |
| **Question** | **Response** | **Mean Age (Years)** | **Mean Farm Experience (Years)** | **Farm Role** | | | **Farm Registration Status** | |
|  |  |  |  | **Owner** | **Manager** | **Other** | **Registered** | **Not Registered** |
| **Do you currently use antibiotics on your farm?** | Yes (%) | 45.1 (25-70) | 13.3 (1-38) | 41 (77.4%) | 10 (18.9%) | 2 (3.8%) | 15 (28.3%) | 38 (71.7%) |
|  | No (%) | 48 (35-70) | 16 (2-40) | 4 (57.1%) | 3 (42.9%) | 0 (0%) | 2 (28.6%) | 5 (71.4%) |
| **Are you aware of the colistin ban in animals in Indonesia?** | Yes (%) | 44.5 (26-68) | 11.9 (3-30) | 8 (72.7%) | 3 (27.3%) | 0 (0%) | 5 (45.5%) | 6 (54.5%) |
|  | No (%) | 50.3 (26-80) | 16.7 (2-40) | 16 (76.2%) | 4 (19%) | 1 (4.8%) | 7 (33.3%) | 14 (66.7%) |
|  | Don't Know (%) | 47.9 (25-70) | 16.2 (1-35) | 19 (76%) | 5 (20%) | 1 (4%) | 8 (32%) | 17 (68%) |
| **Do you use the antibiotic colistin in your layer farm?** | Yes (%) | 0 (0%) | 0 (0%) | 0 (0%) | 0 (0%) | 0 (0%) | 0 (0%) | 0 (0%) |
|  | No (%) | 48.0 (25-80) | 16.4 (1-40) | 50 (80.6%) | 10 (16.1%) | 2 (3.2%) | 19 (30.6%) | 43 (69.4%) |
|  | Don't Know (%) | 43.0 (37-49) | 13.0 (10-20) | 3 (100%) | 0 (0%) | 0 (0%) | 7 (23.3%) | 9 (30%) |
| **Who is responsible for deciding or permitting the use of drugs, especially antibiotics on your farm?** | Veterinarian | 42.0 (39-45) | 2.5 (2-3) | 0 (0%) | 1 (100%) | 0 (0%) | 1 (50%) | 1 (50%) |
|  | Owner | 45.4 (25-70) | 14.5 (1-40) | 33 (89.2%) | 4 (10.8%) | 0 (0%) | 9 (25%) | 22 (75%) |
|  | Farmworker | 68.0 (68-68) | 10.0 (10-10) | 0 (0%) | 0 (0%) | 1 (100%) | 1 (100%) | 0 (0%) |
|  | Other | 43.73 (26-80) | 10.51 (3-40) | 7 (41.2%) | 4 (23.5%) | 6 (35.3%) | 3 (17.6%) | 14 (82.4%) |
| **Based on whose consideration is the use of antibiotics in livestock carried out?** | Veterinarian | 39 (25-70) | 3 (1-40) | 5 (62.5%) | 2 (25%) | 1 (12.5%) | 5 (62.5%) | 10 (37.5%) |
|  | Drug-feed Technical Service | 38 (25-80) | 10 (1-40) | 6 (54.5%) | 3 (27.3%) | 2 (18.2%) | 6 (54.5%) | 8 (45.5%) |
|  | Lesson Learnt | 37 (25-70) | 3 (1-35) | 4 (66.7%) | 2 (33.3%) | 1 (16.7%) | 3 (50%) | 12 (50%) |
|  | Other | 41.86 (26-80) | 12.86 (1-40) | 13 (68.4%) | 4 (21.1%) | 3 (15.8%) | 2 (10.5%) | 14 (73.7%) |
| **Where do the drugs, especially antibiotics used on your farm, come from?** | Farmworker | 39.5 (28-45) | 5.5 (2-10) | 0 (0%) | 0 (0%) | 1 (100%) | 0 (0%) | 1 (100%) |
|  | Owner | 45.5 (25-70) | 13.3 (1-40) | 54 (100%) | 0 (0%) | 0 (0%) | 20 (37%) | 33 (61%) |
|  | Other | 43.93 (25-80) | 10.1 (1-40) | 4 (33.3%) | 0 (0%) | 8 (66.7%) | 1 (8.3%) | 9 (75%) |
|  | With Veterinary Consultation | 46 (29-61) | 11.33 (8-30) | 4 (57.1%) | 0 (0%) | 3 (42.9%) | 3 (42.9%) | 2 (28.6%) |
| *There is no significant difference between demographic variables and the responses to each question. | | | | | | | | |

Fig 1. Number of poultry farms using individual and combination antibiotics before the colistin ban in Indonesia (2013–2016).

Fig. 2. Number of poultry farms using individual and combination antibiotics after the colistin ban in Indonesia (2023). “Not specified” refers to farmer self-reported antimicrobial use for which the specific product name, active ingredient, or antimicrobial class was not provided.
